# Supplementary material for: Is interpregnancy interval associated with cardiovascular risk factors in later life? A cohort study
Source: BMJ Open. 2014 Mar 19;4(3):e004173. doi: 10.1136/bmjopen-2013-004173 (PMC3963084; doi:10.1136/bmjopen-2013-004173)
Supplement: Author's manuscript [file bmjopen-2013-004173.draft_revisions.pdf]

Is inter-pregnancy interval associated with cardiovascular risk factors in later life? : a cohort study

Journal:	*BMJ Open*
Manuscript ID:	bmjopen-2013-004173
Article Type:	Research
Date Submitted by the Author:	04-Oct-2013
Complete List of Authors:	Knipe, Duleeka; University of Bristol, School of Social and Community Medicine<br>Fraser, Abigail; University of Bristol, School of Social and Community Medicine<br>Lawlor, Debbie; University of Bristol, School of Social and Community Medicine<br>Howe, Laura; University of Bristol, Social Medicine
**Primary Subject Heading**:	Epidemiology
Secondary Subject Heading:	Public health
Keywords:	ALSPAC, Inter-pregnancy interval, Cardiovascular disease , Later-life, Women, Pregnancy

SCHOLARONE™
Manuscripts

For peer review only

Is inter-pregnancy interval associated with cardiovascular risk factors in later life? : a cohort study

Duleeka W Knipe, PhD student[1*], Abigail Fraser, research fellow[1,2], Debbie A Lawlor, professor of epidemiology[1,2], Laura D Howe, research fellow[1,2]

[1]School of Social and Community Medicine, University of Bristol, Bristol, United Kingdom

[2]MRC Integrative Epidemiology Unit at the University of Bristol, United Kingdom

This work was carried out at the School of Social and Community Medicine, University of Bristol

*Corresponding author

School of Social and Community Medicine

University of Bristol

Canynge Hall

39 Whatley Road

Bristol

BS8 2PS

Tel. 0117 928 7302

Fax. 0117 928 7325

dee.knipe@bristol.ac.uk

For peer review only

Keywords

ALSPAC

Inter-pregnancy interval

Cardiovascular disease

Later-life

Women

Pregnancy

Word count (excluding title page, abstract, references, figures and tables) **:** 3147

For peer review only

ABSTRACT

Objectives: Pregnancy represents a metabolic challenge to women; in a normal pregnancy, transient metabolic changes occur that support the needs of the growing fetus. It is possible that repeating this challenge within a relatively short amount of time may result in lasting damage to the woman's cardiovascular health. Conversely, it is also possible that long inter-pregnancy intervals may reflect subfertility, which has been found to be associated with cardiovascular disease (CVD). We examine the associations of short and long inter-pregnancy interval with measures of cardiovascular health

Design: Prospective Cohort

Setting: Mothers of the Avon Longitudinal Study of Parents and Children (ALSPAC)

Participants: Women with two live births in order to control for confounding by parity

Outcome Measures: Arterial distensibility, common carotid intima, adiposity, blood pressure, lipids, glucose, insulin, pro-insulin, triglycerides, C-reactive protein

Results: A total of 1268 mothers from the (ALSPAC), who had cardiovascular disease risk factors measured at mean age 48 years. After adjusting for confounding, we found no association of either short (≤15 months) or long (>27 months) inter-pregnancy interval and increased levels of cardiovascular risk factors. There was some suggestion that women with both long and short inter-pregnancy interval had a more favourable lipid profile compared with women whose inter-pregnancy interval was 16-27 months, however the differences were small in magnitude and imprecisely estimated.

Conclusion: This study does not support the hypothesis that either long or short inter-pregnancy interval is a risk factor for later cardiovascular health.

For peer review only - http://bmjopen.bmj.com/site/about/guidelines.xhtml

ARTICLE SUMMARY

Article focus: To examine the associations of short and long inter-pregnancy interval with measures of cardiovascular health

Key Message: This study does not support the hypothesis that either long or short inter-pregnancy interval is a risk factor for later cardiovascular health

Strengths and limitations of this study

Strengths

- Its prospective design with detailed data on reproductive history
- The availability of a wide range of objectively-measured CVD risk factors and the large sample size.
- First study to examine the association of inter-pregnancy interval with cardiovascular risk factors.

Limitations

- As with other prospective cohort studies there was loss to follow-up, with those attending and completing all questionnaires tending to come from a higher socioeconomic background.
- Our analyses was restricted to women who had two live births to remove confounding by parity, and means that our findings may not generalise to women who had three or more children. We did not find any evidence of any strong differences between women who had either only one birth or three or more births, with women with two live/still births
- Inter-pregnancy interval was calculated by subtracting the average gestation period from the birth interval. We do not believe that this would have biased our findings as we used inter-pregnancy interval as a categorical variable and therefore any fluctuations around the average 9 month gestation would not have altered the findings substantially.
- Only generalizable to a largely white European population.
- The population being studied are still young (mean age at clinic =48), it is possible that the association may emerge at older ages, when inter-individual variability in cardiovascular risk factors becomes greater.

For peer review only

INTRODUCTION

Pregnancy represents a metabolic challenge to women; in a normal pregnancy, transient metabolic changes occur that support the needs of the growing foetus. Women become relatively insulin resistant, hyperlipedimic and experience up-regulation of coagulation factors and the inflammatory cascade.[1] A recent systematic review and meta-analysis found that a short inter-pregnancy interval (<12 months) was associated with an increased risk of stillbirths, early neonatal death, preterm birth and low birth weight.[2] Associations between inter-pregnancy interval and cardiovascular outcomes, however, are not known. Given the cardiovascular changes during pregnancy, it is possible that if this challenge is repeated within a relatively short amount of time the effects on a women's metabolic system may be exacerbated and/or be longer lasting. This may have a deleterious effect on her long term cardiovascular health.

Conversely, it is also possible that a long inter-pregnancy interval is associated with increased cardiovascular risk. Longer inter-pregnancy intervals may reflect subfertility, which has been found to be associated with cardiovascular disease (CVD).[3, 4]

Here we examine the associations of short and long inter-pregnancy interval with measures of cardiovascular health (arterial distensibility, common carotid intima, adiposity, blood pressure, lipids, glucose, insulin, pro-insulin, triglycerides, C-reactive protein (CRP)) assessed at a mean age of 48years.

For peer review only - http://bmjopen.bmj.com/site/about/guidelines.xhtml

METHODS

The Avon Longitudinal Study of Parents and Children (ALSPAC) is a prospective population based birth cohort that recruited 14, 541 pregnant women resident in Avon, UK, who had expected delivery dates between 1st April 1991 and 31st December 1992. ALSPAC has previously been described in detail[5], and the study website contains details of all the available data through a fully searchable data dictionary (http://www.bris.ac.uk/alspac/researchers/data-access/data-dictionary). Ethical approval for the study was obtained from the ALSPAC Ethics and Law Committee and the Local Research Ethics Committees.

Pregnancy data and inter-pregnancy interval

In order to remove confounding by parity, which is known to be associated with CVD,[6-12] we restrict our analyses to women with two pregnancies resulting in live births (self-reported).Women were eligible for inclusion in this study if they had two pregnancies that resulted in live births and completed all the questionnaires necessary to ascertain full reproductive history. The study flow diagram is presented in figure 1.

Upon recruitment into the cohort (approximately 18 weeks gestation), women were asked to complete a questionnaire about their health prior to the current pregnancy, including the number of previous pregnancies; the number of miscarriages/abortions; the outcome (live birth, still birth, miscarriage, abortion or termination, live born baby that died, live born baby still alive and other) and end date (i.e. delivery or other outcome) of their most recent previous pregnancy. We used these data, along with subsequent questionnaires (n=5) throughout the 18 year period leading to our outcome assessment to identify women who had two pregnancies that resulted in a live birth. We only included pregnancies that resulted in a live birth and the two pregnancies that we used for the interval could have included those occurring before or after the pregnancy at which women were recruited.

Assessment of cardiovascular risk factors

Between 2009 and 2011 eligible participants (N=11, 264 women) were invited to a research clinic assessment at which a range of cardiovascular outcomes were assessed; this clinic took place between 1.6 and 20.3 years (median 18) since the second birth of the pregnancy interval exposure.

Carotid intima media thickness (cIMT) for both the left and right common carotid artery scans were obtained via high-resolution B ultrasound and imaged longitudinally 1 cm proximal to the carotid bifurcation following a standardized protocol using a ZONARE z.one Ultra convertible ultrasound system with L10-5 linear transducer.

For peer review only - http://bmjopen.bmj.com/site/about/guidelines.xhtml

Images were focused on the posterior (far) wall of the artery and the zoom function was used to magnify the area. Ten-second cine loops were recorded in DICOM format and analysed offline using Carotid Analyser for Research (Vascular Research Tools 5, Medical Imaging Applications, LLC 2008). Three consecutive cardiac cycles were identified and three measures of cIMT were taken from end-diastolic frames and averaged. This was done for both right and left carotid arteries. Arterial distensibility was calculated as the difference between systolic and diastolic arterial diameter. The mean of the left- and right-sided readings was used in analyses. The images were analysed by a single trained reader. Blood pressure was measured while the women were lying down with the use of an Omron M6 monitor (Omron Healthcare UK Ltd, Milton Keynes, UK). Two readings of systolic and diastolic blood pressure were recorded on each arm, and the mean of these 4 readings was used here. Heart rate was measured in both a seated and standing position.

Blood samples were taken after an overnight fast for those attending in the morning or after a minium6 hours fast for those attending the clinic after 14.00. Blood samples were obtained, centrifuged, separated, and frozen at −80°C within 30 minutes. Plasma glucose was measured by automated enzymatic (hexokinase) method. Plasma insulin was measured by an enzyme-linked immunosorbent assay (Mercodia, Uppsala, Sweden) that does not cross-react with proinsulin or c-peptide, and proinsulin was also measured by an enzyme-linked immunosorbent assay (Mercodia) that is a solid-phase 2-site enzyme immunoassay for the quantification of human proinsulin. Lipids were measured by automated analyser with enzymatic methods. CRP was measured by automated particle-enhanced immunoturbidimetric assay (Roche UK, Welwyn Garden City, UK).

Whole body fat mass was measured using dual-energy x-ray absorptiometry (DXA) whole body scans. Weight and height were measured with the subjects in light clothing and without shoes. Weight was measured to the nearest 0.1 kg with the use of Tanita scales. Height was measured to the nearest 0.1 cm with a Harpenden stadiometer. Waist circumference was measured twice to the nearest 1 mm at the midpoint between the lower ribs and the pelvic bone with a flexible tape. The mean of the 2 measures is used here.

A total of 5,005 women attended clinic (44% response). Women attending clinic had a mean age of 48 years Women pregnant at the time of clinic assessment were excluded from our analyses (n=7).

Measurement of Confounding factors

Information on pre-pregnancy weight and height, smoking in pregnancy, single parent households, ethnicity, education, and social class were obtained from questionnaires completed at the time of recruitment during the index

For peer review only - http://bmjopen.bmj.com/site/about/guidelines.xhtml

pregnancy. Ethnicity was categorised as white or non-white. Education was categorized as below or above university education. Household occupational social class was defined according to the 1991 British Office of Population and Census Statistics classification (classes I [professional/managerial] to V [unskilled manual workers]) using either the woman's occupation or her partners, whichever was highest. Smoking in pregnancy was categorized as ever or never smoked during pregnancy, at approximately 18-20 weeks gestation. Ideally, we would wish to measure these potential confounders in relation to the first pregnancy of the interval and for some to have time updated (i.e. also information from the second pregnancy) measurements. In this study, however, we only have data on the pregnancy when women were recruited and this is the first pregnancy of the interval for 30.9% of the women and the second for the remaining 69%.

Statistical Analysis

Distributions of insulin, pro-insulin, triglyceride, CRP, glucose and low-density lipo-protein were right skewed, and so were log transformed for all regression model analyses, which ensured the residuals were approximately normally distributed. We compared women with sufficient data for us to detail their full reproductive history with those women with insufficient data, in terms of all confounding variables. Similarly, we compared those women who formed our eligible sample (only 2 live births and not pregnant at the time of outcome assessment) with women with either only 1 birth or ≥3 more births, in terms of all confounding and outcome variables. Chi-squared tests for categorical data and t-tests for continuous normally distributed variables were performed. Inter-pregnancy interval was calculated as the difference between the dates of delivery of the two pregnancies, minus the average gestation period (9 months). Inter-pregnancy interval was grouped into three categories: ≤15 months, 16-27 and >27 months; these groups correspond to ≤2 years, 2-3 years and >3 years between births.

The association between our categorised measure of inter-pregnancy interval and each outcome was assessed using multiple linear regression, with and without adjustment for potential confounders, comparing both short and long inter-pregnancy interval with the reference category of 16-27 months. All analysis was conducted in Stata/MP 12.0.

Dealing with missing data

Within our eligible study sample of 1268, there was some missing data on potential confounders and outcome measurements (data on exposure had to be observed to be eligible). The extent of missing data varied from 0-29%,

For peer review only - http://bmjopen.bmj.com/site/about/guidelines.xhtml

with blood based measures having the most missing data (web table 5). In order to increase efficiency and minimise selection bias, we imputed missing variables for eligible participants who had missing data on outcomes or confounders, using multivariate multiple imputation. We included all the exposure, confounder and outcome measures in the imputation equations. We used switching regression in Stata and carried out 20 cycles of regression switching and generated 20 imputation datasets. The main analysis results are obtained by averaging across the results from each of these 20 datasets using Rubin's rules and the standard errors for any regression coefficients (used to calculate p-values and 95% confidence intervals) take account of uncertainty in the imputations as well as uncertainty in the estimate.[13] We carried out all our linear regression analysis using both the imputed and complete case datasets. Web table 1 shows that the distributions of variables in the imputed and non-imputed/complete-case datasets were similar.

For peer review only

RESULTS

Sample description

A total of 3451 women had sufficient data to describe a full reproductive history; of these 1970 were excluded because they had either just 1 (n=971) or ≥3 births (n=999). A further 4 were pregnant at the time of assessment and were excluded. Of the remaining 1477 women who had two live births, 147 were excluded as they were recruited into the cohort during their second pregnancy, with the most recent previous pregnancy resulting in a miscarriage, termination, still birth or "other" outcome. Inter-pregnancy interval could be calculated for 1268 of the remaining eligible women (95%). Of these women, 954 attended the clinic assessment in 2009-11 and 792 women had complete data for all outcome and confounder measures (figure 1). Thus, our complete case analysis was performed on 792 women, and analysis using multivariate multiple imputation included 1268 women.

Women who had a provided sufficient data to describe their full reproductive history were on average slightly older, from a higher socioeconomic background, more likely to be smokers and white, and less likely to be in a single parent household, compared with women with insufficient data to describe their full reproductive history (table 1). Women eligible for this study (two live births) were more likely to be smokers compared to women with either 1 or ≥3 births (web table 1). No clear differences with regard to the other confounding variables were observed. There were small differences observed in terms of cardiovascular risk factors (web table 1), with women with single and 3 or more pregnancies having slightly worse cardiovascular risk profile compared to women with 2 pregnancies.

For peer review only - http://bmjopen.bmj.com/site/about/guidelines.xhtml

TABLE 1 – Characteristics of women with full reproductive histories compared to those with incomplete reproductive histories as determined from five questionnaires

	Women with Incomplete Data n=10262	Women with Complete Data n=3451	P-value
Categorical Variables - n (%)			
Ethnicity	n=8653	n=3417	
White	8381 (96.9)	3370 (98.6)	<0.001
Social Class	n=7927	n=3335	
i	842 (10.6)	664 (19.9)	
ii	3099 (39.1)	1601 (48.0)	
iii (non-manual)	2135 (26.9)	738 (22.1)	<0.001
iii (manual)	1261 (15.9)	258 (7.7)	
iv & v	590 (7.4)	74 (2.2)	
Education	n=8739	n=3425	
University Level or above	826 (9.5)	742 (21.7)	<0.001
Single Parent Household	n=9136	n=3403	
Yes	616 (6.7)	102 (3.0)	<0.001
Ever smoked	n=9365	n=3441	
Yes	5124 (54.7)	1369 (39.8)	<0.001
Continuous Variables - mean (SD*)			
Age	n=10258<br>27.4 (5.0)	n=3451<br>29.8 (4.4)	<0.001

* T-test for normally distributed data

For peer review only - http://bmjopen.bmj.com/site/about/guidelines.xhtml

For peer review only

Participant characteristics

The characteristics of women according to their inter-pregnancy interval are presented in table 2. There were no noticeable differences between inter-pregnancy interval categories in terms of pre-pregnancy BMI, ethnicity, social class, education, and smoking status. Women with a longer inter-pregnancy interval, however, were more likely to be in single parent households and slightly younger compared to all other women (table 2). Table 3 presents the distribution of the outcome variables for all those women who attended the follow-up assessment in 2009-11.

For peer review only

For peer review only - http://bmjopen.bmj.com/site/about/guidelines.xhtml

TABLE 2 – Characteristics of women by inter-pregnancy interval N=1268

	Inter-pregnancy interval of all included women n=1268			
	≤15 months *n=269*	16-27 months *n=412*	>27 months *n=582*	p-value
**Continuous Variables - mean (SD)***				
Age at birth of the ALSPAC index child	*n=269*	*n=412*	*n=582*	<0.001
	30.4 (3.7)	30.2 (3.6)	29.3 (4.3)	
Pre-Pregnancy BMI (kg/m2) **	*n=256*	*n=391*	*n=534*	0.6
	22.6 (2.9)	22.5 (3.4)	22.7 (3.4)	
**Categorical Variables - n (%)**				
Ethnicity	*n=267*	*n=411*	*n=577*	0.4
White	265 (99.3)	406 (98.8)	566 (98.1)	
Social Class	*n=261*	*n=409*	*n=568*	0.2
I (highest; professional)	59 (22.6)	89 (21.8)	109 (19.2)	
ii	127 (48.7)	198 (48.4)	274 (48.2)	
iii (non-manual)	54 (20.7)	74 (18.1)	141 (24.8)	
iii (manual)	17 (6.5)	37 (9.1)	34 (6.0)	
iv & v (lowest; unskilled manual)	4 (1.5)	11 (2.7)	10 (1.8)	
Education	*n=269*	*n=411*	*n=580*	0.4
University Level or above	66 (24.5)	82 (20.0)	124 (21.4)	
Single Parent household	*n=269*	*n=412*	*n=578*	<0.001
Yes	1 (0.4)	3 (0.7)	24 (4.2)	
Smoked during pregnancy **	*n=268*	*n=412*	*n=581*	1.0
Yes	101 (38.0)	152 (37.0)	2013 (36.7)	

* SD - Standard Deviation

** Relates to the first child in some women and second in others

For peer review only - http://bmjopen.bmj.com/site/about/guidelines.xhtml

For peer review only

TABLE 3 – Distribution of pre-pregnancy BMI and outcomes at research clinic assessment (2009-11) in the full cohort

Outcome	n=	Mean (SD)*
Age at clinic (years)	4492	47.9 (4.5)
Pre-pregnancy BMI (kg/m2)	4223	22.6 (3.4)
BMI (kg/m2)	4483	26.6 (5.3)
Waist (cm)	4483	84.6 (12.4)
SBP (mm hg)	4357	118.3 (12.9)
DBP (mm hg)	4357	71.8 (8.9)
Heart rate	4357	84 (11.6)
Fat Mass (kg)	4430	27.2 (10893.2)
Avg. Arterial Distensibility (mm)	4408	0.5 (0.1)
Common Carotid Intima (mm)	4415	0.6 (0.1)
Cholesterol (mmol/l)	4252	4.9 (0.9)
High-density Lipoprotein (mmol/l)	4252	1.5 (0.4)
Non-HDL cholesterol (mmol/l)	4252	3.5 (0.9)
		Median (IQR)**
Insulin (mu/1) (log)	4246	4.7 (3.3,7)
Pro-Insulin (pu/l) (log)	4251	5.2 (3.7,7.9)
Triglyceride (mmol/l) (log)	4252	0.9 (0.7,1.2)
C-reactive protein (mg/l) (log)	4252	1 (0.5,2.4)
Glucose (mmol/l) (log)	4252	5.2 (4.9,5.5)

* SD -Standard Deviation

** IQR - Inter-quartile range

For peer review only

Association between inter-pregnancy interval and CVD risk factors

Crude associations of inter-pregnancy interval with outcomes are presented in table 4 and 5. We found no evidence that women with either short or long inter-pregnancy interval had more adverse cardiovascular risk factors compared with women with an inter-pregnancy interval between 16-27 months. There was some suggestion that women with both long and short inter-pregnancy interval had a more favourable lipid profile compared with women whose inter-pregnancy interval was 16-27 months, however the differences were small in magnitude and imprecisely estimated, with the 95% confidence interval including the null value.

Adjustment for potential confounders or for potential mediation by BMI at outcome assessment did not change results (tables 4, 5 and web table 3). These findings are similar to the results when the analysis was restricted to complete cases (web table 4 and 5).

For peer review only

For peer review only - http://bmjopen.bmj.com/site/about/guidelines.xhtml

TABLE 4 – Unadjusted and confounder adjusted analysis of the association between inter-pregnancy interval and blood based cardiovascular risk factors using multivariate multiply imputed data (N=1268)

		Inter-pregnancy interval				
		≤15 months		16-27 months	>27 months	
IBI (months)		β* (95% CI)	P-value	Ref Group	β* (95% CI)	P-value
Cholesterol	Model 1a	-0.1 (-0.2,0.1)	0.3	0	-0.1 (-0.2,0.03)	0.1
(mmol/l)	Model 2b	-0.1 (-0.3,0.1)	0.2	0	-0.05 (-0.2,0.1)	0.4
	Model 1a	0.03 (-0.04,0.1)	0.4	0	-0.01 (-0.1,0.05)	0.8
HDL (mmol/l)	Model 2b	0.03 (-0.04,0.1)	0.4	0	0.01 (0,0.1)	0.6
		% Change (95% CI)	P-value	Ref Group	% Change (95% CI)	P-value
	Model 1a	1 (0.9,1.1)	0.8	0	1.1 (1,1.2)	0.1
Insulin (mu/l)*	Model 2b	1 (0.9,1.1)	0.9	0	1 (1,1.1)	0.4
Pro-Insulin	Model 1a	1 (0.9,1.1)	0.7	0	1.1 (1,1.1)	0.1
(pmol/l)*	Model 2b	1 (0.9,1.1)	0.8	0	1 (1,1.1)	0.2
Triglyceride	Model 1a	0.9 (0.9,1)	0.1	0	0.9 (0.9,1)	0.03
(mmol/l)*	Model 2b	0.9 (0.9,1)	0.1	0	0.9 (0.9,1)	0.02
	Model 1a	0.9 (0.8,1.1)	0.4	0	1.1 (0.9,1.2)	0.4
CRP (mg/l)*	Model 2b	0.9 (0.8,1.1)	0.5	0	1 (0.9,1.2)	0.6
	Model 1a	1 (1,1)	0.7	0	1 (1,1)	0.4
Glucose (mmol/l)*	Model 2b	1 (1,1)	0.6	0	1 (1,1)	0.3

* Mean difference compared with 16-27 months

** Log transformed

[a] Unadjusted model

[b] Adjusted for age, ethnicity, social class, education, pre-pregnancy BMI, ever smoking during pregnancy

For peer review only

TABLE 5 - Unadjusted and confounder adjusted analysis of the association between inter-pregnancy interval and non-blood based cardiovascular risk factors using multivariate multiply imputed data (N=1268)

		Inter-pregnancy interval				
		≤15 months		16-27 months	>27 months	
		β* (95% CI)	P-value	Ref Group	β* (95% CI)	P-value
Avg. Arterial	Model 1[a]	-0.0003 (-0.02,0.02)	1	0	0.02 (0.001,0.04)	0.04
Distensibility (mm)	Model 2[b]	0.002 (-0.02,0.02)	0.9	0	0.01 (-0.003,0.03)	0.1
Common Carotid	Model 1[a]	0 (-0.01,0.02)	0.4	0	-0.0008 (-0.01,0.01)	0.9
Intima (mm)	Model 2[b]	0.003 (-0.01,0.01)	0.6	0	0.002 (-0.01,0.01)	0.7
Total body fat mass	Model 1[a]	-0.2 (-1.7,1.3)	0.8	0	0.4 (-0.8,1.7)	0.5
(kg)	Model 2[b]	-0.3 (-1.6,0.9)	0.6	0	-0.05 (-1.1,1)	0.9
	Model 1[a]	0 (-0.7,0.8)	0.9	0	0.3 (-0.3,0.9)	0.3
BMI (kg/m2)	Model 2[b]	-0.02 (-0.6,0.5)	0.9	0	0 (-0.4,0.5)	0.8
	Model 1[a]	-0.1 (-1.8,1.6)	0.9	0	0.6 (-0.8,2.1)	0.4
Waist (cm)	Model 2[b]	-0.3 (-1.7,1.1)	0.7	0	0.1 (-1.1,1.4)	0.8
	Model 1[a]	0.1 (-1.9,2.2)	0.9	0	-0.6 (-2.3,1.2)	0.5
Systolic BP (mm hg)	Model 2[b]	0.1 (-1.9,2.1)	0.9	0	-0.5 (-2.3,1.2)	0.5
Diastolic BP (mm	Model 1[a]	0.1 (-1.3,1.4)	0.9	0	-0.4 (-1.6,0.8)	0.5
hg)	Model 2[b]	0.1 (-1.2,1.5)	0.9	0	-0.5 (-1.7,0.7)	0.4
	Model 1[a]	0.4 (-1.6,2.4)	0.7	0	0 (-1.7,1.6)	1
Heart Rate	Model 2[b]	0.3 (-1.7,2.4)	0.7	0	0 (-1.6,1.7)	1

* Mean difference compared with 16-27 months

[a] Unadjusted model

[b] Adjusted for age, ethnicity, social class, education, pre-pregnancy BMI, ever smoking during pregnancy

For peer review only

DISCUSSION

We found no association between short or long inter-pregnancy interval and levels of cardiovascular risk factors in a cohort of women with a mean age of 48 years. This is in contrast to our initial hypotheses that shorter inter-pregnancy intervals may give a woman's cardiovascular system insufficient time to recover, that longer inter-pregnancy intervals may reflect subfertility and that both may be associated with adverse levels of cardiovascular risk factors. We actually found weak evidence that women with both short (≤15 months) and long inter-pregnancy intervals (>27) had a more favourable lipid profile (i.e. the opposite of our initial hypotheses) compared with women with an inter-pregnancy interval between 16 and 27 months, but the associations were not large, were imprecisely estimated, and were not consistent across all outcomes and may be due to chance.

To our knowledge, this is the first study to examine the relationship between pregnancy interval and subsequent cardiovascular outcomes. Several previous studies have examined the association of parity with cardiovascular outcomes, with most,[6-12] though not all,[14-16] finding that greater parity is related to more adverse risk factors and greater disease risk. A recent study investigated this association in a population of 1.3 million, using the Swedish registry data,[12] and found a J-shaped relationship between parity and CVD risk, with both nulliparous and grand multiparous (≥ 5 births) having elevated risk compared to those women with 2 births. One possible mechanism for this association is that multiparous women are more likely to have births closer together, and the repeating of the cardiovascular challenge within a relatively short amount of time may lead to the effects on a women's metabolic system to be exacerbated and/or be longer lasting. Our findings do not support this hypothesis. Our results therefore suggest that the association of parity with greater CVD risk may be through another mechanism. Possible theories for the mechanisms underlying the greater risk of CVD after 2 children are: 1) other adverse lifestyle factors being adopted as a family size increases; 2) socio-demographic and other characteristics associated with increased risk of CVD also being associated with having more children; 3) adverse metabolic disturbances accumulating over pregnancies.[12]

The strengths of this study include the prospective design; detailed data on reproductive history; the availability of a wide range of objectively-measured CVD risk factors; and the large sample size. We were able to adjust for a range of potential confounding factors, and we restricted our analyses to women who had two live births in order to remove confounding by parity. To the best of our knowledge, we are the first study to examine the association of inter-pregnancy interval with cardiovascular risk factors. The findings of this study however should be considered

For peer review only - http://bmjopen.bmj.com/site/about/guidelines.xhtml

in light of several limitations. As with other prospective cohort studies there was loss to follow-up, with those attending and completing all questionnaires tending to come from a higher socioeconomic background. Missing data would bias our results if the association between inter-pregnancy interval and cardiovascular risk factors differed in those included in our analyses and those excluded due to missing data. We are unable to test this assumption, but have no reason to suspect that it may be violated. One important consideration when interpreting our results is that we have restricted our analyses to women who had two live births. Whilst we feel that this was a sensible analysis strategy in order to remove confounding by parity, it means that our findings may not generalise to women who had three or more children. We did not find any evidence of any strong differences between women who had either only one birth or three or more births, with women with two live/still births (web table 1). Inter-pregnancy interval was calculated by subtracting the average gestation period (9 months) from the birth interval, ideally we would have liked to have calculated exact gestation periods for each live birth. We do not, however, believe that this would have biased our findings as we used inter-pregnancy interval as a categorical variable and therefore any fluctuations around the average 9 month gestation would not have altered the findings substantially. A further limitation of this study is these findings are only generalizable to a largely white European population. As the population being studied are still young (mean age at clinic =48), it is possible that the association may emerge at older ages, when inter-individual variability in cardiovascular risk factors becomes greater. Ideally we would have measured pre-pregnancy BMI and smoking during pregnancy for the first pregnancy of all women. Due to our study design, this was not possible. These measurements are from the first pregnancy in some women and the second pregnancy in others. There is therefore the possibility that these measurements are not a reasonable representation of levels in the first pregnancy for all women; this may lead to residual confounding. However given that the associations we observe are null, we do not think this has biased our results.

In conclusion, our results do not support an association between inter-pregnancy interval and cardiovascular risk factors. Further studies in different settings such as low income countries where the social patterning of inter-pregnancy interval may differ and in older women with greater inter-individual variability in cardiovascular risk factors would provide a more comprehensive understanding of these associations.

For peer review only

ACKNOWLEDGEMENTS

We are extremely grateful to all the families who took part in this study, the midwives for their help in recruiting them, and the whole ALSPAC team, which includes interviewers, computer and laboratory technicians, clerical workers, research scientists, volunteers, managers, receptionists and nurses. This publication is the work of the authors and DWK will serve as guarantor for the contents of this paper.

COMPETING INTERESTS

None declared

FUNDING

The work was supported by the British Heart Foundation [SP/07/008/24066], the UK Medical Research Council [0701594 to A.F and G1002375 to LH] and the Wellcome Trust (and WT099874MA to D.W.K). AF, DAL and LDH work in a unit that receives funding from the UK Medical Research Council and the University of Bristol. The UK Medical Research Council ([074882], the Wellcome Trust [WT076467] and the University of Bristol provide core funding support for ALSPAC.

AUTHOR CONTRIBUTIONS

DAL, LH and AF conceived the study idea; DWK, LH and AF developed the analysis plan; DWK undertook the analysis under the supervision of LH and AF; DWK wrote the first draft of the paper and collated co-author feedback; and all authors contributed to the critical review and final version.

For peer review only

REFERENCES

1 N. Sattar, Do pregnancy complications and CVD share common antecedents? *Atheroscler Suppl* 2004;**5:**3-7

2 A. Wendt, C. M. Gibbs, S. Peters, et al., Impact of increasing inter-pregnancy interval on maternal and infant health. *Paediatric and perinatal epidemiology* 2012;**26 Suppl 1:**239-58

3 C. G. Solomon, F. B. Hu, A. Dunaif, et al., Menstrual cycle irregularity and risk for future cardiovascular disease. *The Journal of clinical endocrinology and metabolism* 2002;**87:**2013-7

4 N. I. Parikh, S. Cnattingius, M. A. Mittleman, et al., Subfertility and risk of later life maternal cardiovascular disease. *Human reproduction* 2012;**27:**568-75

5 A. Fraser, C. Macdonald-Wallis, K. Tilling, et al., Cohort Profile: The Avon Longitudinal Study of Parents and Children: ALSPAC mothers cohort. *Int J Epidemiol* 2012;97-110

6 F. Atsma, M. L. Bartelink, D. E. Grobbee, et al., Reproductive factors, metabolic factors, and coronary artery calcification in older women. *Menopause* 2008;**15:**899-904

7 J. M. Catov, A. B. Newman, K. Sutton-Tyrrell, et al., Parity and cardiovascular disease risk among older women: how do pregnancy complications mediate the association? *Ann Epidemiol* 2008;**18:**873-9

8 L. G. Gallagher, L. B. Davis, R. M. Ray, et al., Reproductive history and mortality from cardiovascular disease among women textile workers in Shanghai, China. *International Journal of Epidemiology* 2011;**40:**1510-1518

9 A. Green, V. Beral and K. Moser, Mortality in Women in Relation to Their Childbearing History. *Brit Med J* 1988;**297:**391-395

10 D. H. Jaffe, Z. Eisenbach and O. Manor, The effect of parity on cause-specific mortality among married men and women. *Matern Child Health J* 2011;**15:**376-85

11 R. B. Ness, T. Harris, J. Cobb, et al., Number of pregnancies and the subsequent risk of cardiovascular disease. *N Engl J Med* 1993;**328:**1528-33

12 N. I. Parikh, S. Cnattingius, P. W. Dickman, et al., Parity and risk of later-life maternal cardiovascular disease. *American heart journal* 2010;**159:**215-221 e6

13 P. Royston, Multiple imputation of missing values. *Stata J* 2004;**4:**227-241

14 H. S. Chang, N. Odongua, H. Ohrr, et al., Reproductive risk factors for cardiovascular disease mortality among postmenopausal women in Korea: the Kangwha Cohort Study, 1985-2005. *Menopause* 2011;**18:**1205-12

15 G. A. Colditz, W. C. Willett, M. J. Stampfer, et al., A prospective study of age at menarche, parity, age at first birth, and coronary heart disease in women. *Am J Epidemiol* 1987;**126:**861-70

16 K. Steenland, C. Lally and M. Thun, Parity and coronary heart disease among women in the American Cancer Society CPS II population. *Epidemiology* 1996;**7:**641-3

For peer review only

For peer review only - http://bmjopen.bmj.com/site/about/guidelines.xhtml

FIGURES LEGENDS

Figure 1 – Flow-chart of selection of women for analysis

For peer review only

For peer review only - http://bmjopen.bmj.com/site/about/guidelines.xhtml

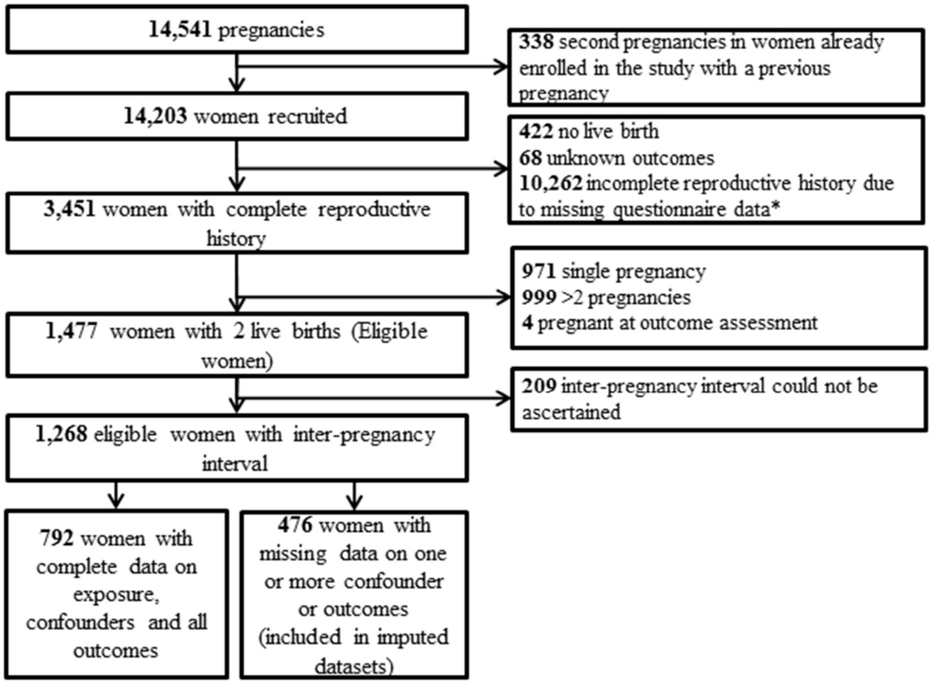

179x124mm (96 x 96 DPI)

view only

Web table 1 – Distributions of imputed characteristics (% or mean (standard error)) in the imputation datasets and in the complete case sample (i.e. non-imputed)

Imputed Variables		Missing (n)	% data imputed	Imputed Data	Non-imputed
**Exposure**					
Inter pregnancy interval		0	0%	N/A	44.85 (0.84)
**Confounders**					
Pre-pregnancy BMI		61	5%	22.6 (0.1)	22.6 (0.1)
Height		17	1%	164.7 (0.2)	164.7 (0.2)
Single parent household		4	0.3%	2%	2%
Education	< O-Level	3	0.2%	13%	13%
	O-Level			34%	34%
	A-Level			32%	32%
	> A-Level			22%	22%
Social Class	i	25	2%	21%	21%
	ii			48%	48%
	iii (non-manual)			22%	22%
	iii (manual)			7%	7%
	iv & v			2%	2%
Ethnicity - White		8	0.6%	99%	99%
Ever Smoked		2	0.2%	37%	37%
Age		0	0%	27.9 (0.1)	27.9 (0.1)
**Outcomes at follow-up clinic**					
Avg. Arterial Distensibility (mm)		323	26%	0.5 (0.004)	0.5 (0.004)
Common Carotid Intima (mm)		323	26%	0.6 (0.002)	0.6 (0.002)
Waist (cm)		324	26%	83.1 (0.3)	82.9 (0.4)
SBP (mm hg)		343	27%	117.9 (0.4)	117.7 (0.4)
Fat Mass (kg)		327	26%	26.3 (293.9)	26.1 (327.9)
BMI (kg/m2)		324	26%	26.1 (0.1)	25.9 (0.2)
DBP (mm hg)		343	27%	71.7 (0.3)	71.5 (0.3)
Heart rate		343	27%	83.7 (0.4)	83.8 (0.4)
Cholesterol (mmol/l)		365	29%	4.9 (0.03)	4.9 (0.03)
HDL (mmol/l)		365	29%	1.5 (0.01)	1.5 (0.01)
LDL (mmol/l)		365	29%	3 (0.03)	3 (0.03)
Non-HDL cholesterol (mmol/l)		365	29%	3.4 (0.03)	3.4 (0.03)
Insulin (mu/1) (log)		370	29%	1.5 (0.02)	1.5 (0.02)
Pro-Insulin (pu/l) (log)		367	29%	1.7 (0.02)	1.7 (0.02)
Triglyceride (mmol/l) (log)		365	29%	-0.1 (0.01)	-0.1 (0.01)
CRP(mg/l) (log)		365	29%	0.1 (0.04)	0 (0.04)
Glucose (mmol/l) (log)		365	29%	1.6 (0.004)	1.6 (0.004)

For peer review only

For peer review only - http://bmjopen.bmj.com/site/about/guidelines.xhtml

Web table 2 – Characteristics of eligible women (with two live births) compared to women with one or three or more live/still births, with full reproductive history n=3447

		No. of births						
		1 birth (n=972)		2 births (n=1479)		3 or more births (n=1000)		P-value
Confounder Variables								
Ethnicity - White n (%)		n=961	948 (98.7)	n=1469	1449 (98.6)	n=987	973 (98.6)	1.0
Social Class n (%)	i		177 (18.9)		297 (20.5)		190 (20.0)	
	ii		471 (50.2)		693 (47.9)		437 (46.1)	
	iii (non-manual)	n=938	224 (23.9)	n=1448	326 (22.5)	n=949	188 (19.8)	<0.001
	iii (manual)		54 (5.8)		100 (6.9)		104 (12.0)	
	iv & v		12 (1.3)		32 (2.2)		30 (3.2)	
Education (University Level or above) n (%)		n=962	212 (22.0)	n=1475	313 (21.2)	n=988	217 (22.0)	0.9
Single Parent Household n (%)		n=946	30 (3.2)	n=1474	35 (2.4)	n=983	37 (3.8)	0.1
Ever smoked n (%)		n=969	380 (39.2)	n=1476	557 (37.7)	n=996	432 (43.4)	0.02
Age mean (SD)*		n=972	29.3 (4.3)	n=1479	29.9 (4.1)	n=1000	30.0 (4.8)	0.0004
Pre-Preg BMI (kg/m2)* - median (IQR)		n=923	21.9 (20.4,24.1)	n=1403	22.1 (20.4,24.1)	n=931	22.1 (20.5,24.1)	0.81
Outcome Variables - median (IQR)								
BMI (kg/m2)*		n=730	25.2 (22.6,28.5)	n=1095	25 (22.6,29.3)	n=705	25.9 (22.9,29.3)	0.003
Waist (cm)*		n=730	81.3 (74.7,89.3)	n=1095	81 (74.8,92.3)	n=705	83.1 (75.7,92.3)	0.0003
SBP (mm hg)*		n=714	116.8 (109.8,126)	n=1071	115.3 (109,125.5)	n=691	117 (110.3,125.5)	0.2
DBP (mm hg)*		n=714	71 (66,76.8)	n=1071	70.3 (65.5,76.3)	n=691	71.3 (66.3,76.3)	1.0
Pulse*		n=714	82.5 (76,89.5)	n=1071	83 (76.8,91.5)	n=691	83.8 (77,91.5)	0.01
Fat Mass (kg)*		n=724	25.3 (19.1,32)	n=1091	24.2 (18.9,34.2)	n=704	25.9 (18.9,34.2)	0.01
Avg. Arterial Distensibility (mm)*		n=716	0.5 (0.4,0.6)	n=1069	0.5 (0.4,0.6)	n=691	0.5 (0.4,0.6)	0.5
Common Carotid Intima (mm)*		n=719	0.6 (0.5,0.6)	n=1069	0.6 (0.5,0.6)	n=691	0.6 (0.5,0.6)	1.0
Cholesterol (mmol/l)*		n=692	4.8 (4.3,5.4)	n=1043	4.9 (4.3,5.5)	n=676	4.9 (4.4,5.5)	0.2
High-density Lipoprotein (mmol/l)*		n=692	1.5 (1.2,1.7)	n=1043	1.5 (1.2,1.7)	n=676	1.4 (1.2,1.7)	0.1
Non-HDL cholesterol (mmol/l)*		n=692	3.3 (2.7,4)	n=1043	3.4 (2.8,4)	n=676	3.5 (2.9,4)	0.1
Very-low density lipoprotein (mmol/l)*		n=692	0.4 (0.3,0.5)	n=1043	0.4 (0.3,0.6)	n=676	0.4 (0.3,0.6)	0.03
Insulin (mu/l)**		n=692	4.7 (3.3,6.3)	n=1038	4.5 (3.2,6.8)	n=674	4.7 (3.2,6.8)	0.2
Pro-insulin (pu/l)**		n=692	5 (3.7,7.2)	n=1041	5 (3.6,8)	n=675	5.3 (3.7,8)	0.2
Triglyceride (mmol/l)**		n=692	0.9 (0.7,1.2)	n=1043	0.9 (0.7,1.2)	n=676	0.9 (0.7,1.2)	0.1
CRP (mg/l)**		n=692	0.9 (0.5,2)	n=1043	0.9 (0.4,2.2)	n=676	1 (0.5,2.2)	0.5
Glucose (mmol/l)**		n=692	5.2 (4.9,5.5)	n=1043	5.1 (4.9,5.5)	n=676	5.2 (4.9,5.5)	0.3

* linear regression for p-value
** log transformed for linear regression

For peer review only

Web table 3 - Confounder and mediator adjusted analysis of the association between inter-pregnancy interval and cardiovascular risk factors using multivariate multiply imputed data (N=1268)

	Inter-pregnancy interval [a]				
	≤15 months		16-27 months	>27 months	
	β* (95% CI)	P-value	Ref Group	β* (95% CI)	P-value
Avg. Arterial Distensibility (mm)	-0.001 (-0.02,0.02)	0.9	0	0.01 (-0.004,0.03)	0.1
Common Carotid Intima (mm)	0.004 (-0.01,0.02)	0.5	0	0.002 (-0.01,0.01)	0.7
Waist (cm)	-0.1 (-1,0.7)	0.8	0	0.1 (-0.7,0.9)	0.8
Systolic BP (mm hg)	0.3 (-1.8,2.3)	0.8	0	-0.4 (-2.2,1.3)	0.6
Diastolic BP (mm hg)	0.2 (-1.1,1.6)	0.8	0	-0.5 (-1.6,0.6)	0.4
Heart Rate	0.4 (-1.5,2.3)	0.7	0	-0.1 (-1.8,1.7)	1.0
Cholesterol (mmol/l)	-0.1 (-0.3,0.04)	0.1	0	-0.1 (-0.2,0.1)	0.4
HDL (mmol/l)	0.02 (-0.05,0.1)	0.6	0	0.02 (-0.04,0.1)	0.6
	% Change (95% CI)	P-value	Ref Group	% Change (95% CI)	P-value
Insulin (mu/l)**	1 (0.9,1.1)	1.0	0	1 (0.9,1.1)	0.6
Pro-Insulin (pmol/l)**	1 (1,1.1)	0.4	0	1.1 (1,1.1)	0.2
Triglyceride (mmol/l)**	0.9 (0.9,1)	0.1	0	0.9 (0.9,1)	0.02
CRP (mg/l)**	0.9 (0.8,1.1)	0.6	0	1 (0.9,1.2)	0.7
Glucose (mmol/l)**	1 (1,1)	0.6	0	1 (1,1)	0.3

* Mean difference compared with 16-27 months

** Log transformed

[a] Adjusted for age, ethnicity, social class, education, pre-pregnancy BMI, ever smoking during pregnancy and BMI at follow-up clinic

For peer review only

Web table 4 – Unadjusted, confounder and mediator adjusted analysis of the association between inter-birth interval and blood based cardiovascular risk factors using complete case data (N=792)

		Inter-pregnancy interval				
		≤15 months		16-27 months	>27 months	
		β* (95% CI)	P-value	Ref Group	β* (95% CI)	P-value
Cholesterol (mmol/l)	Model 1[a]	-0.1 (-0.3,0.04)	0.1	0	-0.1 (-0.3,-0.01)	0.04
	Model 2[b]	-0.2 (-0.3,0.01)	0.06	0	-0.1 (-0.2,0.06)	0.3
	Model 3[c]	-0.2 (-0.3,0)	0.05	0	-0.1 (-0.2,0.06)	0.3
HDL (mmol/l)	Model 1[a]	0.03 (-0.04,0.1)	0.4	0	-0.01 (-0.1,0.05)	0.7
	Model 2[b]	0.02 (-0.05,0.1)	0.5	0	0.02 (-0.03,0.1)	0.4
	Model 3[c]	0.03 (-0.04,0.1)	0.4	0	0.02 (-0.03,0.1)	0.4
		% Change (95% CI)	P-value	Ref Group	% Change (95% CI)	P-value
Insulin (mu/l)**	Model 1[a]	1 (0.9,1.1)	1.0	0	1.1 (1,1.2)	0.1
	Model 2[b]	1 (0.9,1.1)	0.9	0	1 (0.9,1.1)	0.4
	Model 3[c]	1 (0.9,1.1)	1.0	0	1 (1,1.1)	0.4
Pro-Insulin (pmol/l)**	Model 1[a]	1 (0.9,1.2)	0.4	0	1.1 (1,1.2)	0.1
	Model 2[b]	1 (0.9,1.1)	0.5	0	1.1 (1,1.1)	0.2
	Model 3[c]	1 (0.9,1.1)	0.6	0	1.1 (1,1.1)	0.1
Triglyceride (mmol/l)**	Model 1[a]	0.9 (0.9,1)	0.1	0	0.9 (0.9,1)	0.004
	Model 2[b]	0.9 (0.9,1)	0.1	0	0.9 (0.8,1)	0.001
	Model 3[c]	0.9 (0.9,1)	0.0	0	0.9 (0.8,1)	0.001
CRP (mg/l)**	Model 1[a]	0.9 (0.8,1.1)	0.5	0	1.1 (0.9,1.3)	0.4
	Model 2[b]	0.9 (0.8,1.2)	0.6	0	1 (0.8,1.1)	0.7
	Model 3[c]	0.9 (0.8,1.1)	0.4	0	1 (0.8,1.1)	0.7
Glucose (mmol/l)**	Model 1[a]	1 (1,1)	0.6	0	1 (1,1)	0.2
	Model 2[b]	1 (1,1)	0.5	0	1 (1,1)	0.3
	Model 3[c]	1 (1,1)	0.5	0	1 (1,1)	0.3

* Mean difference compared with 16-27 months

** Log transformed

[a] Unadjusted model

[b] Adjusted for age, ethnicity, social class, education, pre-pregnancy BMI, ever smoking during pregnancy

[c] Adjusted for age, ethnicity, social class, education, pre-pregnancy BMI, ever smoking during pregnancy and BMI at follow-up clinic

For peer review only - http://bmjopen.bmj.com/site/about/guidelines.xhtml

Web table 5 - Unadjusted, confounder and mediator analysis of the association between inter-birth interval and non-blood based cardiovascular risk factors using complete case data (N=792)

		Inter-pregnancy interval				
		≤15 months		16-27 months	>27 months	
		β* (95% CI)	P-value	Ref Group	β* (95% CI)	P-value
Avg. Arterial Distensibility (mm)	Model 1[a]	0.002 (-0.02,0.02)	0.8	0	0.02 (0.004,0.04)	0.02
	Model 2[b]	0.002 (-0.02,0.02)	0.8	0	0.01 (-0.004,0.03)	0.1
	Model 3[c]	0.002 (-0.02,0.02)	0.9	0	0.01 (-0.004,0.03)	0.1
Common Carotid Intima (mm)	Model 1[a]	0.01 (-0.005,0.02)	0.3	0	-0.001 (-0.01,0.01)	0.9
	Model 2[b]	0.01 (-0.005,0.02)	0.2	0	0.01 (-0.005,0.01)	0.3
	Model 3[c]	0.01 (-0.005,0.02)	0.3	0	0.005 (-0.005,0.01)	0.3
Total body fat mass (kg)	Model 1[a]	0.02 (-1.8,1.8)	1.0	0	0.6 (-0.8,2.1)	0.4
	Model 2[b]	-0.4 (-1.7,1)	0.6	0	-0.3 (-1.4,0.8)	0.6
BMI (kg/m2)	Model 1[a]	0.1 (-0.7,0.9)	0.8	0	0.4 (-0.3,1.1)	0.3
	Model 2[b]	0 (-0.5,0.5)	1.0	0	0 (-0.5,0.4)	0.9
Waist (cm)	Model 1[a]	0.01 (-2,2)	1.0	0	0.8 (-0.8,2.4)	0.3
	Model 2[b]	-0.2 (-1.7,1.3)	0.8	0	0.3 (-1,1.5)	0.7
	Model 3[c]	-0.2 (-1.1,0.7)	0.7	0	0.3 (-0.4,1.1)	0.4
Systolic BP (mm hg)	Model 1[a]	0.3 (-1.9,2.6)	0.8	0	-0.4 (-2.2,1.5)	0.7
	Model 2[b]	0.2 (-2.1,2.4)	0.9	0	-0.7 (-2.6,1.2)	0.4
	Model 3[c]	0.1 (-2.1,2.3)	0.9	0	-0.8 (-2.6,1.1)	0.4
Diastolic BP (mm hg)	Model 1[a]	0.2 (-1.3,1.6)	0.8	0	-0.2 (-1.5,1)	0.7
	Model 2[b]	0.01 (-1.5,1.5)	1.0	0	-0.6 (-1.9,0.6)	0.3
	Model 3[c]	-0.02 (-1.5,1.5)	1.0	0	-0.6 (-1.9,0.6)	0.3
Heart Rate	Model 1[a]	0.5 (-1.4,2.5)	0.6	0	-0.02 (-1.6,1.6)	1.0
	Model 2[b]	0.1 (-1.9,2.1)	0.9	0	-0.3 (-2.1,1.4)	0.7
	Model 3[c]	0.1 (-1.9,2.1)	0.9	0	-0.3 (-2,1.4)	0.7

* Mean difference compared with 16-27 months

[a] Unadjusted model

[b] Adjusted for l age, l ethnicity, social class, education, pre-pregnancy BMI, ever smoking during pregnancy

[c] Adjusted for age, ethnicity, social class, education, pre-pregnancy BMI, ever smoking during pregnancy and BMI at follow-up clinic

For peer review only

For peer review only

For peer review only - http://bmjopen.bmj.com/site/about/guidelines.xhtml

STROBE 2007 (v4) Statement—Checklist of items that should be included in reports of *cohort studies*

Section/Topic	Item #	Recommendation	Reported on page #
**Title and abstract**	1	(*a*) Indicate the study's design with a commonly used term in the title or the abstract	1
		(*b*) Provide in the abstract an informative and balanced summary of what was done and what was found	3
**Introduction**			
Background/rationale	2	Explain the scientific background and rationale for the investigation being reported	5
Objectives	3	State specific objectives, including any prespecified hypotheses	5
**Methods**			
Study design	4	Present key elements of study design early in the paper	6
Setting	5	Describe the setting, locations, and relevant dates, including periods of recruitment, exposure, follow-up, and data collection	6-8
Participants	6	(*a*) Give the eligibility criteria, and the sources and methods of selection of participants. Describe methods of follow-up	6-8 & figure 1
		(*b*) For matched studies, give matching criteria and number of exposed and unexposed	n/a
Variables	7	Clearly define all outcomes, exposures, predictors, potential confounders, and effect modifiers. Give diagnostic criteria, if applicable	6-8
Data sources/ measurement	8*	For each variable of interest, give sources of data and details of methods of assessment (measurement). Describe comparability of assessment methods if there is more than one group	*6-8*
Bias	9	Describe any efforts to address potential sources of bias	17-18 & web tables
Study size	10	Explain how the study size was arrived at	6-9 & figure 1
Quantitative variables	11	Explain how quantitative variables were handled in the analyses. If applicable, describe which groupings were chosen and why	8
Statistical methods	12	(*a*) Describe all statistical methods, including those used to control for confounding	8-9
		(*b*) Describe any methods used to examine subgroups and interactions	8-9
		(*c*) Explain how missing data were addressed	8-9
		(*d*) If applicable, explain how loss to follow-up was addressed	17
		(*e*) Describe any sensitivity analyses	8-9
**Results**			

For peer review only

Participants	13*	(a) Report numbers of individuals at each stage of study—eg numbers potentially eligible, examined for eligibility, confirmed eligible, included in the study, completing follow-up, and analysed	9 & Figure 1
		(b) Give reasons for non-participation at each stage	9 & Figure 1
		(c) Consider use of a flow diagram	Figure 1
Descriptive data	14*	(a) Give characteristics of study participants (eg demographic, clinical, social) and information on exposures and potential confounders	10 & 12
		(b) Indicate number of participants with missing data for each variable of interest	Web table
		(c) Summarise follow-up time (eg, average and total amount)	13
Outcome data	15*	Report numbers of outcome events or summary measures over time	13
Main results	16	(*a*) Give unadjusted estimates and, if applicable, confounder-adjusted estimates and their precision (eg, 95% confidence interval). Make clear which confounders were adjusted for and why they were included	
		(*b*) Report category boundaries when continuous variables were categorized	15 & 16
		(*c*) If relevant, consider translating estimates of relative risk into absolute risk for a meaningful time period	
Other analyses	17	Report other analyses done—eg analyses of subgroups and interactions, and sensitivity analyses	14 & web tables
**Discussion**			
Key results	18	Summarise key results with reference to study objectives	17
**Limitations**			
Interpretation	20	Give a cautious overall interpretation of results considering objectives, limitations, multiplicity of analyses, results from similar studies, and other relevant evidence	17-18
Generalisability	21	Discuss the generalisability (external validity) of the study results	17-18
**Other information**			
Funding	22	Give the source of funding and the role of the funders for the present study and, if applicable, for the original study on which the present article is based	19

*Give information separately for cases and controls in case-control studies and, if applicable, for exposed and unexposed groups in cohort and cross-sectional studies.

Note: An Explanation and Elaboration article discusses each checklist item and gives methodological background and published examples of transparent reporting. The STROBE checklist is best used in conjunction with this article (freely available on the Web sites of PLoS Medicine at http://www.plosmedicine.org/, Annals of Internal Medicine at http://www.annals.org/, and Epidemiology at http://www.epidem.com/). Information on the STROBE Initiative is available at www.strobe-statement.org.

For peer review only

Is inter-pregnancy interval associated with cardiovascular risk factors in later life? : a cohort study

Journal:	*BMJ Open*
Manuscript ID:	bmjopen-2013-004173.R1
Article Type:	Research
Date Submitted by the Author:	24-Jan-2014

Complete List of Authors:	Knipe, Duleeka; University of Bristol, School of Social and Community Medicine<br>Fraser, Abigail; University of Bristol, School of Social and Community Medicine<br>Lawlor, Debbie; University of Bristol, School of Social and Community Medicine<br>Howe, Laura; University of Bristol, Social Medicine

**Primary Subject Heading**:	Epidemiology
Secondary Subject Heading:	Public health
Keywords:	ALSPAC, Inter-pregnancy interval, Cardiovascular disease , Later-life, Women, Pregnancy

SCHOLARONE™ Manuscripts

For peer review only

Is inter-pregnancy interval associated with cardiovascular risk factors in later life? : a cohort study

Duleeka W Knipe, PhD student[1*], Abigail Fraser, research fellow[1,2], Debbie A Lawlor, professor of epidemiology[1,2], Laura D Howe, research fellow[1,2]

[1]School of Social and Community Medicine, University of Bristol, Bristol, United Kingdom

[2]MRC Integrative Epidemiology Unit at the University of Bristol, United Kingdom

This work was carried out at the School of Social and Community Medicine, University of Bristol

*Corresponding author

School of Social and Community Medicine

University of Bristol

Canynge Hall

39 Whatley Road

Bristol

BS8 2PS

Tel. 0117 928 7302

Fax. 0117 928 7325

dee.knipe@bristol.ac.uk

For peer review only

For peer review only - http://bmjopen.bmj.com/site/about/guidelines.xhtml

Keywords

ALSPAC

Inter-pregnancy interval

Cardiovascular disease

Later-life

Women

Pregnancy

Word count (excluding title page, abstract, references, figures and tables) **:** 3147

For peer review only

ABSTRACT

Objectives: Pregnancy represents a metabolic challenge to women; in a normal pregnancy, transient metabolic changes occur that support the needs of the growing fetus. It is possible that repeating this challenge within a relatively short amount of time may result in lasting damage to the woman's cardiovascular health. Conversely, it is also possible that long inter-pregnancy intervals (IPI) may reflect subfertility, which has been found to be associated with cardiovascular disease (CVD). We examine the associations of short and long IPI with measures of cardiovascular health.

Design: Prospective Cohort

Setting: Mothers of the Avon Longitudinal Study of Parents and Children (ALSPAC)

Participants: Women with two live births in order to control for confounding by parity

Outcome Measures: Arterial distensibility, common carotid intima, adiposity, blood pressure, lipids, glucose, insulin, pro-insulin, triglycerides, C-reactive protein

Results: Twenty five percent (n=3451) of ALSPAC mothers had provided sufficient data to determine full reproductive history - of these 1477, had two live births, with 54% mothers having non-missing data on all variables required for our analyses. A total of 1268 mothers with IPI (inter-birth interval minus 9 months gestation) had cardiovascular disease risk factors measured/imputed at mean age 48 years. After adjusting for confounding, we found no association of either short (≤15 months) or long (>27 months) IPI and increased levels of cardiovascular risk factors. There was some suggestion that women with both long and short IPI had a more favourable lipid profile compared with women whose IPI was 16-27 months, however the differences were small in magnitude and imprecisely estimated.

Conclusion: This study does not support the hypothesis that either long or short IPI is a risk factor for later cardiovascular health.

For peer review only - http://bmjopen.bmj.com/site/about/guidelines.xhtml

ARTICLE SUMMARY

Article focus: To examine the associations of short and long inter-pregnancy interval with measures of cardiovascular health

Key Message: This study does not support the hypothesis that either long or short inter-pregnancy interval is a risk factor for later cardiovascular health

Strengths and limitations of this study

Strengths

- Its prospective design with detailed data on reproductive history
- The availability of a wide range of objectively-measured CVD risk factors and the large sample size.
- First study to examine the association of inter-pregnancy interval (IPI) with cardiovascular risk factors.

Limitations

- As with other prospective cohort studies there was loss to follow-up, with those attending and completing all questionnaires tending to come from a higher socioeconomic background.
- Our analyses was restricted to women who had two live births to remove confounding by parity, and means that our findings may not generalise to women who had three or more children. We did not find any evidence of any strong differences between women who had either only one birth or three or more births, with women with two live/still births
- IPI was calculated by subtracting the average gestation period from the birth interval. We do not believe that this would have biased our findings as we used IPI as a categorical variable and therefore any fluctuations around the average 9 month gestation would not have altered the findings substantially.
- Only generalizable to a largely white European population.
- The population being studied are still young (mean age at clinic =48), it is possible that the association may emerge at older ages, when inter-individual variability in cardiovascular risk factors becomes greater.

For peer review only - http://bmjopen.bmj.com/site/about/guidelines.xhtml

INTRODUCTION

Pregnancy represents a metabolic challenge to women; in a normal pregnancy, transient metabolic changes occur that support the needs of the growing foetus. Women become relatively insulin resistant, hyperlipedimic and experience up-regulation of coagulation factors and the inflammatory cascade.[1] A recent systematic review and meta-analysis found that a short inter-pregnancy interval (IPI) (<12 months) was associated with an increased risk of stillbirths, early neonatal death, preterm birth and low birth weight.[2] Associations between IPI and cardiovascular outcomes, however, are not known. Given the cardiovascular changes during pregnancy, it is possible that if this challenge is repeated within a relatively short amount of time the effects on a women's metabolic system may be exacerbated and/or be longer lasting. This may have a deleterious effect on her long term cardiovascular health.

Conversely, it is also possible that a long IPI is associated with increased cardiovascular risk. Longer IPIs may reflect subfertility, which has been found to be associated with cardiovascular disease (CVD).[3, 4]

Here we examine the associations of short and long IPI with measures of cardiovascular health (arterial distensibility, common carotid intima, adiposity, blood pressure, lipids, glucose, insulin, pro-insulin, triglycerides, C-reactive protein (CRP)) assessed at a mean age of 48years.

For peer review only

METHODS

The Avon Longitudinal Study of Parents and Children (ALSPAC) is a prospective population based birth cohort that recruited 14, 541 pregnant women resident in Avon, UK, who had expected delivery dates between 1st April 1991 and 31st December 1992. ALSPAC has previously been described in detail[5], and the study website contains details of all the available data through a fully searchable data dictionary (http://www.bris.ac.uk/alspac/researchers/data-access/data-dictionary). Ethical approval for the study was obtained from the ALSPAC Ethics and Law Committee and the Local Research Ethics Committees.

Pregnancy data and IPI

In order to remove confounding by parity, which is known to be associated with CVD,[6-12] we restrict our analyses to women with two pregnancies resulting in live births (self-reported).Women were eligible for inclusion in this study if they had two pregnancies that resulted in live births and completed all the questionnaires necessary to ascertain full reproductive history. The study flow diagram is presented in figure 1.

Upon recruitment into the cohort (approximately 18 weeks gestation), women were asked to complete a questionnaire about their health prior to the current pregnancy, including the number of previous pregnancies; the number of miscarriages/abortions; the outcome (live birth, still birth, miscarriage, abortion or termination, live born baby that died, live born baby still alive and other) and end date (i.e. delivery or other outcome) of their most recent previous pregnancy. We used these data, along with subsequent questionnaires (n=5) throughout the 18 year period leading to our outcome assessment to identify women who had two pregnancies that resulted in a live birth. We only included pregnancies that resulted in a live birth and the two pregnancies that we used for the interval could have included those occurring before or after the pregnancy at which women were recruited.

Assessment of cardiovascular risk factors

Between 2009 and 2011 ALSPAC mothers (N=11, 264 women) were invited to a research clinic assessment at which a range of cardiovascular outcomes were assessed; this clinic took place between 1.6 and 20.3 years (median 18) since the second birth defining the end of the IPI. Not all of these women were eligible for inclusion in our analysis as not all of them had full reproductive histories recorded.

Carotid intima media thickness (cIMT) for both the left and right common carotid artery scans were obtained via high-resolution B ultrasound and imaged longitudinally 1 cm proximal to the carotid bifurcation following a

For peer review only - http://bmjopen.bmj.com/site/about/guidelines.xhtml

standardized protocol using a ZONARE z.one Ultra convertible ultrasound system with L10-5 linear transducer. Images were focused on the posterior (far) wall of the artery and the zoom function was used to magnify the area. Ten-second cine loops were recorded in DICOM format and analysed offline using Carotid Analyser for Research (Vascular Research Tools 5, Medical Imaging Applications, LLC 2008). Three consecutive cardiac cycles were identified and three measures of cIMT were taken from end-diastolic frames and averaged. This was done for both right and left carotid arteries. Arterial distensibility was calculated as the difference between systolic and diastolic arterial diameter. The mean of the left- and right-sided readings was used in analyses. The images were analysed by a single trained reader. Blood pressure was measured while the women were lying down with the use of an Omron M6 monitor (Omron Healthcare UK Ltd, Milton Keynes, UK). Two readings of systolic and diastolic blood pressure were recorded on each arm, and the mean of these 4 readings was used here. Heart rate was measured in both a seated and standing position.

Blood samples were taken after an overnight fast for those attending in the morning or after a minium6 hours fast for those attending the clinic after 14.00. Blood samples were obtained, centrifuged, separated, and frozen at −80°C within 30 minutes. Plasma glucose was measured by automated enzymatic (hexokinase) method. Plasma insulin was measured by an enzyme-linked immunosorbent assay (Mercodia, Uppsala, Sweden) that does not cross-react with proinsulin or c-peptide, and proinsulin was also measured by an enzyme-linked immunosorbent assay (Mercodia) that is a solid-phase 2-site enzyme immunoassay for the quantification of human proinsulin. Lipids were measured by automated analyser with enzymatic methods. CRP was measured by automated particle-enhanced immunoturbidimetric assay (Roche UK, Welwyn Garden City, UK).

Whole body fat mass was measured using dual-energy x-ray absorptiometry (DXA) whole body scans. Weight and height were measured with the subjects in light clothing and without shoes. Weight was measured to the nearest 0.1 kg with the use of Tanita scales. Height was measured to the nearest 0.1 cm with a Harpenden stadiometer. Waist circumference was measured twice to the nearest 1 mm at the midpoint between the lower ribs and the pelvic bone with a flexible tape. The mean of the 2 measures is used here.

A total of 4834 women attended clinic (43% response)[5]. Women attending clinic had a mean age of 48 years Women pregnant at the time of clinic assessment were excluded from our analyses (n=7). Only 954 women attending clinic and not pregnant at the time of clinic assessment, had full reproductive histories.

Measurement of Confounding factors

For peer review only - http://bmjopen.bmj.com/site/about/guidelines.xhtml

Information on pre-pregnancy weight and height, smoking in pregnancy, single parent households, ethnicity, education, and social class were obtained from questionnaires completed at the time of recruitment during the index pregnancy. Ethnicity was categorised as white or non-white. Education was categorized as below or above university education. Household occupational social class was defined according to the 1991 British Office of Population and Census Statistics classification (classes I [professional/managerial] to V [unskilled manual workers]) using either the woman's occupation or her partners, whichever was highest. Smoking in pregnancy was categorized as ever or never smoked during pregnancy, at approximately 18-20 weeks gestation. Ideally, we would wish to measure these potential confounders in relation to the first pregnancy of the interval and for some to have time updated (i.e. also information from the second pregnancy) measurements. In this study, however, we only have data on the pregnancy when women were recruited and this is the first pregnancy of the interval for 30.9% of the women and the second for the remaining 69%. Participant's age at clinic attendance was included as a confounding factor.

Statistical Analysis

Distributions of insulin, pro-insulin, triglyceride, CRP, glucose and low-density lipo-protein were right skewed, and so were log transformed for all regression model analyses, which ensured the residuals were approximately normally distributed. We compared women with sufficient data for us to detail their full reproductive history with those women with insufficient data, in terms of all confounding variables. Similarly, we compared those women who formed our eligible sample (only 2 live births and not pregnant at the time of outcome assessment) with women with either only 1 birth or ≥3 more births, in terms of all confounding and outcome variables. Chi-squared tests for categorical data and t-tests for continuous normally distributed variables were performed. IPI was calculated as the difference between the dates of delivery of the two pregnancies, minus the average gestation period (9 months). IPI was grouped into three categories: ≤15 months, 16-27 and >27 months; these groups correspond to ≤2 years, 2-3 years and >3 years between births.

The association between our categorised measure of IPI and each outcome was assessed using multiple linear regression, with and without adjustment for potential confounders, comparing both short and long IPI with the reference category of 16-27 months. All analysis was conducted in Stata/MP 12.0.

In order to assess whether our results were biased as a result of some confounders being measured at the first pregnancy of the IPI for some women and the second for others, we stratified our results by whether the ALSPAC child was the first or second pregnancy of the interval.

For peer review only - http://bmjopen.bmj.com/site/about/guidelines.xhtml

Dealing with missing data

Within our eligible study sample of 1268, there was some missing data on potential confounders and outcome measurements (data on exposure had to be observed to be eligible). The extent of missing data varied from 0-29%, with blood based measures having the most missing data (web table 1). In order to increase efficiency and minimise selection bias, we imputed missing variables for eligible participants who had missing data on outcomes or confounders, using multivariate multiple imputation. We included all the exposure, confounder and outcome measures in the imputation equations. We used switching regression in Stata and carried out 20 cycles of regression switching and generated 20 imputation datasets. The main analysis results are obtained by averaging across the results from each of these 20 datasets using Rubin's rules and the standard errors for any regression coefficients (used to calculate p-values and 95% confidence intervals) take account of uncertainty in the imputations as well as uncertainty in the estimate.[13] We carried out all our linear regression analysis using both the imputed and complete case datasets. Web table 1 shows that the distributions of variables in the imputed and non-imputed/complete-case datasets were similar.

RESULTS

Sample description

A total of 3451 women out of 13,713 recruited women who had a live birth had sufficient data to describe a full reproductive history; of these 1970 were excluded because they had either just 1 (n=971) or ≥3 births (n=999). A further 4 were pregnant at the time of assessment and were excluded. Of the remaining 1477 women who had two live births, 147 were excluded as they were recruited into the cohort during their second pregnancy, with the most recent previous pregnancy resulting in a miscarriage, termination, still birth or "other" outcome. IPI could be calculated for 1268 of the remaining eligible women (95%). Of these women, 954 attended the clinic assessment in 2009-11 and 792 women had complete data for all outcome and confounder measures (figure 1). Thus, our complete case analysis was performed on 792 women, and analysis using multivariate multiple imputation included 1268 women. Women included in the complete case analysis versus women with missing data on covariates and/or outcome variables had similar IPI (web table 2).

Women who had a provided sufficient data to describe their full reproductive history were on average slightly older, from a higher socioeconomic background, more likely to be smokers and white, and less likely to be in a single parent household, compared with women with insufficient data to describe their full reproductive history (table 1).

For peer review only - http://bmjopen.bmj.com/site/about/guidelines.xhtml

Women eligible for this study (two live births) were more likely to be smokers compared to women with either 1 or ≥3 births (web table 3). No clear differences with regard to the other confounding variables were observed. There were small differences observed in terms of cardiovascular risk factors (web table 3), with women with single and 3 or more pregnancies having slightly worse cardiovascular risk profile compared to women with 2 pregnancies.

For peer review only

For peer review only - http://bmjopen.bmj.com/site/about/guidelines.xhtml

TABLE 1 – Characteristics of women with full reproductive histories compared to those with incomplete reproductive histories as determined from five questionnaires

	Women with Incomplete Data n=10262	Women with Complete Data n=3451	P-value
Categorical Variables - n (%)			
Ethnicity	n=8653	n=3417	
White	8381 (96.9)	3370 (98.6)	<0.001
Social Class	n=7927	n=3335	
i	842 (10.6)	664 (19.9)	
ii	3099 (39.1)	1601 (48.0)	
iii (non-manual)	2135 (26.9)	738 (22.1)	<0.001
iii (manual)	1261 (15.9)	258 (7.7)	
iv & v	590 (7.4)	74 (2.2)	
Education	n=8739	n=3425	
University Level or above	826 (9.5)	742 (21.7)	<0.001
Single Parent Household	n=9136	n=3403	
Yes	616 (6.7)	102 (3.0)	<0.001
Ever smoked	n=9365	n=3441	
Yes	5124 (54.7)	1369 (39.8)	<0.001
Continuous Variables - mean (SD*)			
Age at birth of the ALSPAC	n=10258	n=3451	<0.001
index child	27.4 (5.0)	29.8 (4.4)	

* T-test for normally distributed data

For peer review only - http://bmjopen.bmj.com/site/about/guidelines.xhtml

Participant characteristics

The characteristics of women according to their IPI are presented in table 2. There were no noticeable differences between IPI categories in terms of pre-pregnancy BMI, ethnicity, social class, education, and smoking status. Women with a longer IPI, however, were more likely to be in single parent households and slightly younger compared to all other women (table 2). Table 3 presents the distribution of the outcome variables for all those women who attended the follow-up assessment in 2009-11 and who had an IPI calculated for this analysis.

For peer review only

For peer review only - http://bmjopen.bmj.com/site/about/guidelines.xhtml

TABLE 2 – Characteristics of women by inter-pregnancy interval N=1268

	Inter-pregnancy interval of all included women n=1268			
	≤15 months	16-27 months	>27 months	
	n=269	*n=412*	*n=582*	p-value
**Continuous Variables - mean (SD)***				
Age at birth of the ALSPAC index child	*n=269*	*n=412*	*n=582*	<0.001
	30.4 (3.7)	30.2 (3.6)	29.3 (4.3)	
Pre-Pregnancy BMI (kg/m2) **	*n=256*	*n=391*	*n=534*	0.6
	22.6 (2.9)	22.5 (3.4)	22.7 (3.4)	
**Categorical Variables - n (%)**				
Ethnicity	*n=267*	*n=411*	*n=577*	0.4
White	265 (99.3)	406 (98.8)	566 (98.1)	
Social Class	*n=261*	*n=409*	*n=568*	0.2
I (highest; professional)	59 (22.6)	89 (21.8)	109 (19.2)	
ii	127 (48.7)	198 (48.4)	274 (48.2)	
iii (non-manual)	54 (20.7)	74 (18.1)	141 (24.8)	
iii (manual)	17 (6.5)	37 (9.1)	34 (6.0)	
iv & v (lowest; unskilled manual)	4 (1.5)	11 (2.7)	10 (1.8)	
Education	*n=269*	*n=411*	*n=580*	0.4
University Level or above	66 (24.5)	82 (20.0)	124 (21.4)	
Single Parent household	*n=269*	*n=412*	*n=578*	<0.001
Yes	1 (0.4)	3 (0.7)	24 (4.2)	
Smoked during pregnancy **	*n=268*	*n=412*	*n=581*	1.0
Yes	101 (38.0)	152 (37.0)	2013 (36.7)	

* SD - Standard Deviation

** Relates to the first child in some women and second in others

For peer review only - http://bmjopen.bmj.com/site/about/guidelines.xhtml

For peer review only

TABLE 3 – Distribution of pre-pregnancy BMI and outcomes at research clinic assessment (2009-11) with IPI

	Inter-pregnancy interval n=1268					
		≤15 months		16-27 months		>27 months
	n=	*n=271*	*n=*	*n=415*	*n=*	*n=582*
**Mean (Standard deviation)**						
Pre-pregnancy BMI (kg/m2)	260	22.6(2.8)	399	22.5(3.4)	553	22.7(3.4)
BMI (kg/m2)	203	25.9(4.5)	313	25.8(4.7)	433	26.1(4.8)
Waist (cm)	203	82.6(11.4)	313	82.6(11.2)	433	83.3(11.2)
SBP (mm hg)	202	118.1(12.5)	304	118(12.3)	424	117.4(12.5)
DBP (mm hg)	202	71.7(8.3)	304	71.7(8.5)	424	71.4(8.1)
Heart rate	202	84.3(11.1)	304	83.7(10.7)	424	83.6(11)
Fat Mass (kg)	204	26(10.1)	314	25.9(9.9)	428	26.3(10.1)
Avg. Arterial Distensibility (mm)	204	0.5(0.1)	315	0.5(0.1)	430	0.5(0.1)
Common Carotid Intima (mm)	204	0.6(0.1)	315	0.6(0.1)	430	0.6(0.1)
Cholesterol (mmol/l)	194	4.9(0.8)	303	5(0.9)	410	4.9(0.9)
High-density Lipoprotein (mmol/l)	194	1.5(0.4)	303	1.5(0.4)	410	1.5(0.4)
Non-HDL cholesterol (mmol/l)	194	3.4(0.9)	303	3.5(0.9)	410	3.4(0.9)
**Median (Inter-quartile range)**						
Insulin (mu/1)	193	4.3(3.2,6.4)	301	4.3(3,6.3)	408	4.6(3.3,6.8)
Pro-Insulin (pu/l)	194	5(3.6,7.3)	302	4.9(3.5,6.6)	409	5.1(3.6,7.9)
Triglyceride (mmol/l)	194	0.8(0.6,1.1)	303	0.9(0.7,1.2)	410	0.9(0.7,1.1)
C-reactive protein (mg/l)	194	0.9(0.4,1.9)	303	0.9(0.5,2)	410	1(0.5,2.5)
Glucose (mmol/l)	194	5.2(4.9,5.4)	303	5.2(4.9,5.4)	410	5.1(4.9,5.4)

For peer review only

For peer review only - http://bmjopen.bmj.com/site/about/guidelines.xhtml

Association between IPI and CVD risk factors

Crude associations of IPI with outcomes are presented in table 4 and 5. We found no evidence that women with either short or long IPI had more adverse cardiovascular risk factors compared with women with an IPI between 16-27 months. There was some suggestion that women with both long and short IPI had a more favourable lipid profile compared with women whose IPI was 16-27 months, however the differences were small in magnitude and imprecisely estimated, with the 95% confidence interval including the null value.

Adjustment for potential confounders or for potential mediation by BMI at outcome assessment did not change results (tables 4, 5 and web table 4). These findings are similar to the results when the analysis was restricted to complete cases (web table 5 and 6). The results were also similar when the women were stratified according to whether the ALSPAC child was the first or second pregnancy (results not shown – available from authors on request)

For peer review only

TABLE 4 – Unadjusted and confounder adjusted analysis of the association between inter-pregnancy interval and blood based cardiovascular risk factors using multivariate multiply imputed data (N=1268)

		Inter-pregnancy interval				
		≤15 months		16-27 months	>27 months	
	Analysis Model Number	β* (95% CI)	P-value	Ref Group	β* (95% CI)	P-value
Cholesterol (mmol/l)	Model 1[a]	-0.1 (-0.2,0.1)	0.3	0	-0.1 (-0.2,0.03)	0.1
	Model 2[b]	-0.1 (-0.3,0.1)	0.2	0	-0.05 (-0.2,0.1)	0.4
HDL (mmol/l)	Model 1[a]	0.03 (-0.04,0.1)	0.4	0	-0.01 (-0.1,0.05)	0.8
	Model 2[b]	0.03 (-0.04,0.1)	0.4	0	0.01 (0,0.1)	0.6
		% Change (95% CI)	P-value	Ref Group	% Change (95% CI)	P-value
Insulin (mu/l)**	Model 1[a]	1 (0.9,1.1)	0.8	0	1.1 (1,1.2)	0.1
	Model 2[b]	1 (0.9,1.1)	0.9	0	1 (1,1.1)	0.4
Pro-Insulin (pmol/l)**	Model 1[a]	1 (0.9,1.1)	0.7	0	1.1 (1,1.1)	0.1
	Model 2[b]	1 (0.9,1.1)	0.8	0	1 (1,1.1)	0.2
Triglyceride (mmol/l)**	Model 1[a]	0.9 (0.9,1)	0.1	0	0.9 (0.9,1)	0.03
	Model 2[b]	0.9 (0.9,1)	0.1	0	0.9 (0.9,1)	0.02
CRP (mg/l)**	Model 1[a]	0.9 (0.8,1.1)	0.4	0	1.1 (0.9,1.2)	0.4
	Model 2[b]	0.9 (0.8,1.1)	0.5	0	1 (0.9,1.2)	0.6
Glucose (mmol/l)**	Model 1[a]	1 (1,1)	0.7	0	1 (1,1)	0.4
	Model 2[b]	1 (1,1)	0.6	0	1 (1,1)	0.3

* Mean difference compared with 16-27 months

** Log transformed

[a] Unadjusted model

[b] Adjusted for maternal age, maternal ethnicity, social class, education, pre-pregnancy BMI, ever smoking during pregnancy

For peer review only

TABLE 5 - Unadjusted and confounder adjusted analysis of the association between inter-pregnancy interval and non-blood based cardiovascular risk factors using multivariate multiply imputed data (N=1268)

	Analysis Model Number	Inter-pregnancy interval ≤15 months β* (95% CI)	P-value	16-27 months Ref Group	>27 months β* (95% CI)	P-value
Avg. Arterial Distensibility (mm)	Model 1[a]	-0.0003 (-0.02,0.02)	1	0	0.02 (0.001,0.04)	0.04
	Model 2[b]	0.002 (-0.02,0.02)	0.9	0	0.01 (-0.003,0.03)	0.1
Common Carotid Intima (mm)	Model 1[a]	0.005 (-0.01,0.02)	0.4	0	-0.0008 (-0.01,0.01)	0.9
	Model 2[b]	0.003 (-0.01,0.01)	0.6	0	0.002 (-0.01,0.01)	0.7
Total body fat mass (kg)[c]	Model 1[a]	-0.2 (-1.7,1.3)	0.8	0	0.4 (-0.8,1.7)	0.5
	Model 2[b]	-0.3 (-1.6,0.9)	0.6	0	-0.05 (-1.1,1)	0.9
BMI (kg/m2)	Model 1[a]	0.03 (-0.7,0.8)	0.9	0	0.3 (-0.3,0.9)	0.3
	Model 2[b]	-0.02 (-0.6,0.5)	0.9	0	0 (-0.4,0.5)	0.8
Waist (cm)	Model 1[a]	-0.1 (-1.8,1.6)	0.9	0	0.6 (-0.8,2.1)	0.4
	Model 2[b]	-0.3 (-1.7,1.1)	0.7	0	0.1 (-1.1,1.4)	0.8
Systolic BP (mm hg)	Model 1[a]	0.1 (-1.9,2.2)	0.9	0	-0.6 (-2.3,1.2)	0.5
	Model 2[b]	0.1 (-1.9,2.1)	0.9	0	-0.5 (-2.3,1.2)	0.5
Diastolic BP (mm hg)	Model 1[a]	0.1 (-1.3,1.4)	0.9	0	-0.4 (-1.6,0.8)	0.5
	Model 2[b]	0.1 (-1.2,1.5)	0.9	0	-0.5 (-1.7,0.7)	0.4
Heart Rate	Model 1[a]	0.4 (-1.6,2.4)	0.7	0	0 (-1.7,1.6)	1
	Model 2[b]	0.3 (-1.7,2.4)	0.7	0	0 (-1.6,1.7)	1

* Mean difference compared with 16-27 months

[a] Unadjusted model

[b] Adjusted for age, ethnicity, social class, education, pre-pregnancy BMI, ever smoking during pregnancy

[c] Height and height^2 were included into the model

For peer review only

DISCUSSION

We found no association between short or long IPI and levels of cardiovascular risk factors in a cohort of women with a mean age of 48 years. This is in contrast to our initial hypotheses that shorter IPIs may give a woman's cardiovascular system insufficient time to recover, that longer IPIs may reflect subfertility and that both may be associated with adverse levels of cardiovascular risk factors. We actually found weak evidence that women with both short (≤15 months) and long IPIs (>27) had a more favourable lipid profile (i.e. the opposite of our initial hypotheses) compared with women with an IPI between 16 and 27 months, but the associations were not large, were imprecisely estimated, and were not consistent across all outcomes and may be due to chance.

To our knowledge, this is the first study to examine the relationship between pregnancy interval and subsequent cardiovascular outcomes. Several previous studies have examined the association of parity with cardiovascular outcomes, with most,[6-12] though not all,[14-16] finding that greater parity is related to more adverse risk factors and greater disease risk. A recent study investigated this association in a population of 1.3 million, using the Swedish registry data,[12] and found a J-shaped relationship between parity and CVD risk, with both nulliparous and grand multiparous (≥ 5 births) having elevated risk compared to those women with 2 births. One possible mechanism for this association is that multiparous women are more likely to have births closer together, and the repeating of the cardiovascular challenge within a relatively short amount of time may lead to the effects on a women's metabolic system to be exacerbated and/or be longer lasting. Our findings do not support this hypothesis. Our results therefore suggest that the association of parity with greater CVD risk may be through another mechanism. Possible theories for the mechanisms underlying the greater risk of CVD after 2 children are: 1) other adverse lifestyle factors being adopted as a family size increases; 2) socio-demographic and other characteristics associated with increased risk of CVD also being associated with having more children; 3) adverse metabolic disturbances accumulating over pregnancies.[12]

The strengths of this study include the prospective design; detailed data on reproductive history; the availability of a wide range of objectively-measured CVD risk factors; and the large sample size. We were able to adjust for a range of potential confounding factors, and we restricted our analyses to women who had two live births in order to remove confounding by parity. To the best of our knowledge, we are the first study to examine the association of IPI with cardiovascular risk factors. The findings of this study however should be considered in light of several limitations. As with other prospective cohort studies there was loss to follow-up, with those attending and completing all questionnaires tending to come from a higher socioeconomic background. This means that the women included in

For peer review only - http://bmjopen.bmj.com/site/about/guidelines.xhtml

our analysis are not a representative sample of the full ALSPAC cohort; some studies suggest that lack of generalisability does not necessarily result in selection bias [17-20], but we cannot be certain of this from the current analysis and replication of our results in other studies with different distributions of socio-demographic variables would be beneficial. Missing data would bias our results if the association between IPI and cardiovascular risk factors differed in those included in our analyses and those excluded due to missing data. We are unable to test this assumption, but have no reason to suspect that it may be violated. One important consideration when interpreting our results is that we have restricted our analyses to women who had two live births. Whilst we feel that this was a sensible analysis strategy in order to remove confounding by parity, it means that our findings may not generalise to women who had three or more children. We did not find any evidence of any strong differences between women who had either only one birth or three or more births, with women with two live/still births (web table 3). IPI was calculated by subtracting the average gestation period (9 months) from the birth interval, ideally we would have liked to have calculated exact gestation periods for each live birth. We do not, however, believe that this would have biased our findings as we used IPI as a categorical variable and therefore any fluctuations around the average 9 month gestation would not have altered the findings substantially. It is possible that by calculating our IPI in this way we have attenuated our results towards the null. A further limitation of this study is these findings are only generalizable to a largely white European population with a higher socioeconomic status. As the population being studied are still young (mean age at clinic =48), it is possible that the association may emerge at older ages, when inter-individual variability in cardiovascular risk factors becomes greater. Ideally we would have measured pre-pregnancy BMI and smoking during pregnancy for the first pregnancy of all women. Due to our study design, this was not possible. These measurements are from the first pregnancy in some women and the second pregnancy in others. There is therefore the possibility that these measurements are not a reasonable representation of levels in the first pregnancy for all women; this may lead to residual confounding. However given that the associations we observe are null, we do not think this has biased our results.

In conclusion, our results do not support an association between IPI and cardiovascular risk factors, though our findings must be interpreted in light of the large losses to follow-up and limitations in our measurement of IPI. Further studies in other populations with more detailed data on gestational age at delivery of all pregnancies, in different settings such as low income countries where the social patterning of IPI may differ and in older women with greater inter-individual variability in cardiovascular risk factors would provide a more comprehensive understanding of these associations.

For peer review only - http://bmjopen.bmj.com/site/about/guidelines.xhtml

For peer review only

For peer review only

For peer review only - http://bmjopen.bmj.com/site/about/guidelines.xhtml

ACKNOWLEDGEMENTS

We are extremely grateful to all the families who took part in this study, the midwives for their help in recruiting them, and the whole ALSPAC team, which includes interviewers, computer and laboratory technicians, clerical workers, research scientists, volunteers, managers, receptionists and nurses. This publication is the work of the authors and DWK will serve as guarantor for the contents of this paper.

COMPETING INTERESTS

None declared

FUNDING

The work was supported by the British Heart Foundation [SP/07/008/24066], the UK Medical Research Council [0701594 to A.F and G1002375 to LH] and the Wellcome Trust (and WT099874MA to D.W.K). AF, DAL and LDH work in a unit that receives funding from the UK Medical Research Council and the University of Bristol. The UK Medical Research Council ([074882], the Wellcome Trust [WT076467] and the University of Bristol provide core funding support for ALSPAC.

AUTHOR CONTRIBUTIONS

DAL, LH and AF conceived the study idea; DWK, LH and AF developed the analysis plan; DWK undertook the analysis under the supervision of LH and AF; DWK wrote the first draft of the paper and collated co-author feedback; and all authors contributed to the critical review and final version.

DATA SHARING

This study is based on the ALSPAC study. ALSPAC has a detailed data sharing policy which can be found at : http://www.bristol.ac.uk/alspac/researchers/data-access/policy/

For peer review only

REFERENCES

1 N. Sattar, Do pregnancy complications and CVD share common antecedents? *Atheroscler Suppl* 2004;**5:**3-7

2 A. Wendt, C. M. Gibbs, S. Peters, et al., Impact of increasing inter-pregnancy interval on maternal and infant health. *Paediatric and perinatal epidemiology* 2012;**26 Suppl 1:**239-58

3 C. G. Solomon, F. B. Hu, A. Dunaif, et al., Menstrual cycle irregularity and risk for future cardiovascular disease. *The Journal of clinical endocrinology and metabolism* 2002;**87:**2013-7

4 N. I. Parikh, S. Cnattingius, M. A. Mittleman, et al., Subfertility and risk of later life maternal cardiovascular disease. *Human reproduction* 2012;**27:**568-75

5 A. Fraser, C. Macdonald-Wallis, K. Tilling, et al., Cohort Profile: The Avon Longitudinal Study of Parents and Children: ALSPAC mothers cohort. *Int J Epidemiol* 2012;97-110

6 F. Atsma, M. L. Bartelink, D. E. Grobbee, et al., Reproductive factors, metabolic factors, and coronary artery calcification in older women. *Menopause* 2008;**15:**899-904

7 J. M. Catov, A. B. Newman, K. Sutton-Tyrrell, et al., Parity and cardiovascular disease risk among older women: how do pregnancy complications mediate the association? *Ann Epidemiol* 2008;**18:**873-9

8 L. G. Gallagher, L. B. Davis, R. M. Ray, et al., Reproductive history and mortality from cardiovascular disease among women textile workers in Shanghai, China. *International Journal of Epidemiology* 2011;**40:**1510-1518

9 A. Green, V. Beral and K. Moser, Mortality in Women in Relation to Their Childbearing History. *Brit Med J* 1988;**297:**391-395

10 D. H. Jaffe, Z. Eisenbach and O. Manor, The effect of parity on cause-specific mortality among married men and women. *Matern Child Health J* 2011;**15:**376-85

11 R. B. Ness, T. Harris, J. Cobb, et al., Number of pregnancies and the subsequent risk of cardiovascular disease. *N Engl J Med* 1993;**328:**1528-33

12 N. I. Parikh, S. Cnattingius, P. W. Dickman, et al., Parity and risk of later-life maternal cardiovascular disease. *American heart journal* 2010;**159:**215-221 e6

13 P. Royston, Multiple imputation of missing values. *Stata J* 2004;**4:**227-241

14 H. S. Chang, N. Odongua, H. Ohrr, et al., Reproductive risk factors for cardiovascular disease mortality among postmenopausal women in Korea: the Kangwha Cohort Study, 1985-2005. *Menopause* 2011;**18:**1205-12

15 G. A. Colditz, W. C. Willett, M. J. Stampfer, et al., A prospective study of age at menarche, parity, age at first birth, and coronary heart disease in women. *Am J Epidemiol* 1987;**126:**861-70

16 K. Steenland, C. Lally and M. Thun, Parity and coronary heart disease among women in the American Cancer Society CPS II population. *Epidemiology* 1996;**7:**641-3

17 A. J. Sogaard, R. Selmer, E. Bjertness, et al., The Oslo Health Study: The impact of self-selection in a large, population-based survey. *International journal for equity in health* 2004;**3:**3

18 A. J. Van Loon, M. Tijhuis, H. S. Picavet, et al., Survey non-response in the Netherlands: effects on prevalence estimates and associations. *Ann Epidemiol* 2003;**13:**105-10

19 R. M. Nilsen, S. E. Vollset, H. K. Gjessing, et al., Self-selection and bias in a large prospective pregnancy cohort in Norway. *Paediatric and perinatal epidemiology* 2009;**23:**597-608

20 E. A. Nohr, M. Frydenberg, T. B. Henriksen, et al., Does low participation in cohort studies induce bias? *Epidemiology* 2006;**17:**413-8

For peer review only

For peer review only - http://bmjopen.bmj.com/site/about/guidelines.xhtml

FIGURES LEGENDS

Figure 1 – Flow-chart of selection of women for analysis

For peer review only

For peer review only - http://bmjopen.bmj.com/site/about/guidelines.xhtml

Is inter-pregnancy interval associated with cardiovascular risk factors in later life? : a cohort study

Duleeka W Knipe, PhD student[1*], Abigail Fraser, research fellow[1,2], Debbie A Lawlor, professor of epidemiology[1,2], Laura D Howe, research fellow[1,2]

[1]School of Social and Community Medicine, University of Bristol, Bristol, United Kingdom

[2]MRC Integrative Epidemiology Unit at the University of Bristol, United Kingdom

This work was carried out at the School of Social and Community Medicine, University of Bristol

*Corresponding author

School of Social and Community Medicine

University of Bristol

Canynge Hall

39 Whatley Road

Bristol

BS8 2PS

Tel. 0117 928 7302

Fax. 0117 928 7325

dee.knipe@bristol.ac.uk

For peer review only

Keywords

ALSPAC

Inter-pregnancy interval

Cardiovascular disease

Later-life

Women

Pregnancy

Word count (excluding title page, abstract, references, figures and tables) **:** 3147

For peer review only

ABSTRACT

Objectives: Pregnancy represents a metabolic challenge to women; in a normal pregnancy, transient metabolic changes occur that support the needs of the growing fetus. It is possible that repeating this challenge within a relatively short amount of time may result in lasting damage to the woman's cardiovascular health. Conversely, it is also possible that long inter-pregnancy intervals (IPI) may reflect subfertility, which has been found to be associated with cardiovascular disease (CVD). We examine the associations of short and long ~~inter-pregnancy interval~~IPI with measures of cardiovascular health.

Design: Prospective Cohort

Setting: Mothers of the Avon Longitudinal Study of Parents and Children (ALSPAC)

Participants: Women with two live births in order to control for confounding by parity

Outcome Measures: Arterial distensibility, common carotid intima, adiposity, blood pressure, lipids, glucose, insulin, pro-insulin, triglycerides, C-reactive protein

Results: Twenty five percent (n=3451) of ALSPAC mothers had provided sufficient data to determine full reproductive history - of these 1477, had two live births, with 54% mothers having non-missing data on all variables required for our analyses. A total of 1268 mothers with IPI (inter-birth interval minus 9 months gestation) ~~from the (ALSPAC), who~~ had cardiovascular disease risk factors measured/imputed at mean age 48 years. After adjusting for confounding, we found no association of either short (≤15 months) or long (>27 months) ~~inter-pregnancy interval~~IPI and increased levels of cardiovascular risk factors. There was some suggestion that women with both long and short ~~inter-pregnancy interval~~IPI had a more favourable lipid profile compared with women whose ~~inter-pregnancy interval~~IPI was 16-27 months, however the differences were small in magnitude and imprecisely estimated.

Conclusion: This study does not support the hypothesis that either long or short ~~inter-pregnancy interval~~IPI is a risk factor for later cardiovascular health.

For peer review only

For peer review only - http://bmjopen.bmj.com/site/about/guidelines.xhtml

ARTICLE SUMMARY

Article focus: To examine the associations of short and long inter-pregnancy interval with measures of cardiovascular health

Key Message: This study does not support the hypothesis that either long or short inter-pregnancy interval is a risk factor for later cardiovascular health

Strengths and limitations of this study

Strengths

- Its prospective design with detailed data on reproductive history
- The availability of a wide range of objectively-measured CVD risk factors and the large sample size.
- First study to examine the association of inter-pregnancy interval (IPI) with cardiovascular risk factors.

Limitations

- As with other prospective cohort studies there was loss to follow-up, with those attending and completing all questionnaires tending to come from a higher socioeconomic background.
- Our analyses was restricted to women who had two live births to remove confounding by parity, and means that our findings may not generalise to women who had three or more children. We did not find any evidence of any strong differences between women who had either only one birth or three or more births, with women with two live/still births
- ~~Inter-pregnancy interval~~IPI was calculated by subtracting the average gestation period from the birth interval. We do not believe that this would have biased our findings as we used ~~inter-pregnancy interval~~IPI as a categorical variable and therefore any fluctuations around the average 9 month gestation would not have altered the findings substantially.
- Only generalizable to a largely white European population.
- The population being studied are still young (mean age at clinic =48), it is possible that the association may emerge at older ages, when inter-individual variability in cardiovascular risk factors becomes greater.

For peer review only

INTRODUCTION

Pregnancy represents a metabolic challenge to women; in a normal pregnancy, transient metabolic changes occur that support the needs of the growing foetus. Women become relatively insulin resistant, hyperlipedimic and experience up-regulation of coagulation factors and the inflammatory cascade.[1] A recent systematic review and meta-analysis found that a short inter-pregnancy interval (IPI) (<12 months) was associated with an increased risk of stillbirths, early neonatal death, preterm birth and low birth weight.[2] Associations between inter-pregnancy intervalIPI and cardiovascular outcomes, however, are not known. Given the cardiovascular changes during pregnancy, it is possible that if this challenge is repeated within a relatively short amount of time the effects on a women's metabolic system may be exacerbated and/or be longer lasting. This may have a deleterious effect on her long term cardiovascular health.

Conversely, it is also possible that a long inter-pregnancy intervalIPI is associated with increased cardiovascular risk. Longer inter-pregnancy intervalIPIs may reflect subfertility, which has been found to be associated with cardiovascular disease (CVD).[3, 4]

Here we examine the associations of short and long inter-pregnancy intervalIPI with measures of cardiovascular health (arterial distensibility, common carotid intima, adiposity, blood pressure, lipids, glucose, insulin, pro-insulin, triglycerides, C-reactive protein (CRP)) assessed at a mean age of 48years.

For peer review only - http://bmjopen.bmj.com/site/about/guidelines.xhtml

METHODS

The Avon Longitudinal Study of Parents and Children (ALSPAC) is a prospective population based birth cohort that recruited 14, 541 pregnant women resident in Avon, UK, who had expected delivery dates between 1st April 1991 and 31st December 1992. ALSPAC has previously been described in detail[5], and the study website contains details of all the available data through a fully searchable data dictionary (http://www.bris.ac.uk/alspac/researchers/data-access/data-dictionary). Ethical approval for the study was obtained from the ALSPAC Ethics and Law Committee and the Local Research Ethics Committees.

Pregnancy data and ~~inter-pregnancy interval~~IPI

In order to remove confounding by parity, which is known to be associated with CVD,[6-12] we restrict our analyses to women with two pregnancies resulting in live births (self-reported).Women were eligible for inclusion in this study if they had two pregnancies that resulted in live births and completed all the questionnaires necessary to ascertain full reproductive history. The study flow diagram is presented in figure 1.

Upon recruitment into the cohort (approximately 18 weeks gestation), women were asked to complete a questionnaire about their health prior to the current pregnancy, including the number of previous pregnancies; the number of miscarriages/abortions; the outcome (live birth, still birth, miscarriage, abortion or termination, live born baby that died, live born baby still alive and other) and end date (i.e. delivery or other outcome) of their most recent previous pregnancy. We used these data, along with subsequent questionnaires (n=5) throughout the 18 year period leading to our outcome assessment to identify women who had two pregnancies that resulted in a live birth. We only included pregnancies that resulted in a live birth and the two pregnancies that we used for the interval could have included those occurring before or after the pregnancy at which women were recruited.

Assessment of cardiovascular risk factors

Between 2009 and 2011 ALSPAC mothers ~~eligible participants~~ (N=11, 264 women) were invited to a research clinic assessment at which a range of cardiovascular outcomes were assessed; this clinic took place between 1.6 and 20.3 years (median 18) since the second birth defining the end of the ~~pregnancy interval exposure~~IPI. Not all of these women were eligible for inclusion in our analysis as not all of them had full reproductive histories recorded.

Carotid intima media thickness (cIMT) for both the left and right common carotid artery scans were obtained via high-resolution B ultrasound and imaged longitudinally 1 cm proximal to the carotid bifurcation following a

For peer review only - http://bmjopen.bmj.com/site/about/guidelines.xhtml

standardized protocol using a ZONARE z.one Ultra convertible ultrasound system with L10-5 linear transducer. Images were focused on the posterior (far) wall of the artery and the zoom function was used to magnify the area. Ten-second cine loops were recorded in DICOM format and analysed offline using Carotid Analyser for Research (Vascular Research Tools 5, Medical Imaging Applications, LLC 2008). Three consecutive cardiac cycles were identified and three measures of cIMT were taken from end-diastolic frames and averaged. This was done for both right and left carotid arteries. Arterial distensibility was calculated as the difference between systolic and diastolic arterial diameter. The mean of the left- and right-sided readings was used in analyses. The images were analysed by a single trained reader. Blood pressure was measured while the women were lying down with the use of an Omron M6 monitor (Omron Healthcare UK Ltd, Milton Keynes, UK). Two readings of systolic and diastolic blood pressure were recorded on each arm, and the mean of these 4 readings was used here. Heart rate was measured in both a seated and standing position.

Blood samples were taken after an overnight fast for those attending in the morning or after a minium6 hours fast for those attending the clinic after 14.00. Blood samples were obtained, centrifuged, separated, and frozen at −80°C within 30 minutes. Plasma glucose was measured by automated enzymatic (hexokinase) method. Plasma insulin was measured by an enzyme-linked immunosorbent assay (Mercodia, Uppsala, Sweden) that does not cross-react with proinsulin or c-peptide, and proinsulin was also measured by an enzyme-linked immunosorbent assay (Mercodia) that is a solid-phase 2-site enzyme immunoassay for the quantification of human proinsulin. Lipids were measured by automated analyser with enzymatic methods. CRP was measured by automated particle-enhanced immunoturbidimetric assay (Roche UK, Welwyn Garden City, UK).

Whole body fat mass was measured using dual-energy x-ray absorptiometry (DXA) whole body scans. Weight and height were measured with the subjects in light clothing and without shoes. Weight was measured to the nearest 0.1 kg with the use of Tanita scales. Height was measured to the nearest 0.1 cm with a Harpenden stadiometer. Waist circumference was measured twice to the nearest 1 mm at the midpoint between the lower ribs and the pelvic bone with a flexible tape. The mean of the 2 measures is used here.

A total of 4834~~5,005~~ women attended clinic (434% response)[5].~~.~~ Women attending clinic had a mean age of 48 years Women pregnant at the time of clinic assessment were excluded from our analyses (n=7). Only 954 women attending clinic and not pregnant at the time of clinic assessment, had full reproductive histories.

Measurement of Confounding factors

For peer review only - http://bmjopen.bmj.com/site/about/guidelines.xhtml

For peer review only

Information on pre-pregnancy weight and height, smoking in pregnancy, single parent households, ethnicity, education, and social class were obtained from questionnaires completed at the time of recruitment during the index pregnancy. Ethnicity was categorised as white or non-white. Education was categorized as below or above university education. Household occupational social class was defined according to the 1991 British Office of Population and Census Statistics classification (classes I [professional/managerial] to V [unskilled manual workers]) using either the woman's occupation or her partners, whichever was highest. Smoking in pregnancy was categorized as ever or never smoked during pregnancy, at approximately 18-20 weeks gestation. Ideally, we would wish to measure these potential confounders in relation to the first pregnancy of the interval and for some to have time updated (i.e. also information from the second pregnancy) measurements. In this study, however, we only have data on the pregnancy when women were recruited and this is the first pregnancy of the interval for 30.9% of the women and the second for the remaining 69%. -Participant's age at clinic attendance was included as a confounding factor.

Statistical Analysis

Distributions of insulin, pro-insulin, triglyceride, CRP, glucose and low-density lipo-protein were right skewed, and so were log transformed for all regression model analyses, which ensured the residuals were approximately normally distributed. We compared women with sufficient data for us to detail their full reproductive history with those women with insufficient data, in terms of all confounding variables. Similarly, we compared those women who formed our eligible sample (only 2 live births and not pregnant at the time of outcome assessment) with women with either only 1 birth or ≥3 more births, in terms of all confounding and outcome variables. Chi-squared tests for categorical data and t-tests for continuous normally distributed variables were performed. ~~Inter-pregnancy interval~~IPI was calculated as the difference between the dates of delivery of the two pregnancies, minus the average gestation period (9 months). ~~Inter-pregnancy interval~~IPI was grouped into three categories: ≤15 months, 16-27 and >27 months; these groups correspond to ≤2 years, 2-3 years and >3 years between births.

The association between our categorised measure of ~~inter-pregnancy interval~~IPI and each outcome was assessed using multiple linear regression, with and without adjustment for potential confounders, comparing both short and long ~~inter-pregnancy interval~~IPI with the reference category of 16-27 months. All analysis was conducted in Stata/MP 12.0.

For peer review only

In order to assess whether our results were biased as a result of some confounders being measured at the first pregnancy of the IPI for some women and the second for others, we stratified our results by whether the ALSPAC child was the first or second pregnancy of the interval.

Dealing with missing data

Within our eligible study sample of 1268, there was some missing data on potential confounders and outcome measurements (data on exposure had to be observed to be eligible). The extent of missing data varied from 0-29%, with blood based measures having the most missing data (web table 15). In order to increase efficiency and minimise selection bias, we imputed missing variables for eligible participants who had missing data on outcomes or confounders, using multivariate multiple imputation. We included all the exposure, confounder and outcome measures in the imputation equations. We used switching regression in Stata and carried out 20 cycles of regression switching and generated 20 imputation datasets. The main analysis results are obtained by averaging across the results from each of these 20 datasets using Rubin's rules and the standard errors for any regression coefficients (used to calculate p-values and 95% confidence intervals) take account of uncertainty in the imputations as well as uncertainty in the estimate.[13] We carried out all our linear regression analysis using both the imputed and complete case datasets. Web table 1 shows that the distributions of variables in the imputed and non-imputed/complete-case datasets were similar.

RESULTS

Sample description

A total of 3451 women out of 13,713 recruited women who had a live birth had sufficient data to describe a full reproductive history; of these 1970 were excluded because they had either just 1 (n=971) or ≥3 births (n=999). A further 4 were pregnant at the time of assessment and were excluded. Of the remaining 1477 women who had two live births, 147 were excluded as they were recruited into the cohort during their second pregnancy, with the most recent previous pregnancy resulting in a miscarriage, termination, still birth or "other" outcome. ~~Inter-pregnancy interval~~IPI could be calculated for 1268 of the remaining eligible women (95%). Of these women, 954 attended the clinic assessment in 2009-11 and 792 women had complete data for all outcome and confounder measures (figure 1). Thus, our complete case analysis was performed on 792 women, and analysis using multivariate multiple imputation included 1268 women. Women included in the complete case analysis versus women with missing data on covariates and/or outcome variables had similar IPI (web table 2).

For peer review only - http://bmjopen.bmj.com/site/about/guidelines.xhtml

Women who had a provided sufficient data to describe their full reproductive history were on average slightly older, from a higher socioeconomic background, more likely to be smokers and white, and less likely to be in a single parent household, compared with women with insufficient data to describe their full reproductive history (table 1). Women eligible for this study (two live births) were more likely to be smokers compared to women with either 1 or ≥3 births (web table ~~1~~3). No clear differences with regard to the other confounding variables were observed. There were small differences observed in terms of cardiovascular risk factors (web table ~~1~~3), with women with single and 3 or more pregnancies having slightly worse cardiovascular risk profile compared to women with 2 pregnancies.

For peer review only

For peer review only - http://bmjopen.bmj.com/site/about/guidelines.xhtml

TABLE 1 – Characteristics of women with full reproductive histories compared to those with incomplete reproductive histories as determined from five questionnaires

	Women with Incomplete Data n=10262	Women with Complete Data n=3451	P-value
Categorical Variables - n (%)			
Ethnicity	n=8653	n=3417	
White	8381 (96.9)	3370 (98.6)	<0.001
Social Class	n=7927	n=3335	
i	842 (10.6)	664 (19.9)	
ii	3099 (39.1)	1601 (48.0)	
iii (non-manual)	2135 (26.9)	738 (22.1)	<0.001
iii (manual)	1261 (15.9)	258 (7.7)	
iv & v	590 (7.4)	74 (2.2)	
Education	n=8739	n=3425	
University Level or above	826 (9.5)	742 (21.7)	<0.001
Single Parent Household	n=9136	n=3403	
Yes	616 (6.7)	102 (3.0)	<0.001
Ever smoked	n=9365	n=3441	
Yes	5124 (54.7)	1369 (39.8)	<0.001
Continuous Variables - mean (SD*)			
~~Age~~ Age at birth of the ALSPAC index child	n=10258<br>27.4 (5.0)	n=3451<br>29.8 (4.4)	<0.001

* T-test for normally distributed data

For peer review only

Participant characteristics

The characteristics of women according to their ~~inter-pregnancy interval~~IPI are presented in table 2. There were no noticeable differences between ~~inter-pregnancy interval~~IPI categories in terms of pre-pregnancy BMI, ethnicity, social class, education, and smoking status. Women with a longer ~~inter-pregnancy interval~~IPI, however, were more likely to be in single parent households and slightly younger compared to all other women (table 2). Table 3 presents the distribution of the outcome variables for all those women who attended the follow-up assessment in 2009-11 and who had an IPI calculated for this analysis.~~.~~

For peer review only

For peer review only - http://bmjopen.bmj.com/site/about/guidelines.xhtml

TABLE 2 – Characteristics of women by inter-pregnancy interval N=1268

	Inter-pregnancy interval of all included women n=1268			
	≤15 months *n=269*	16-27 months *n=412*	>27 months *n=582*	p-value
**Continuous Variables - mean (SD)***				
Age at birth of the ALSPAC index child	*n=269* 30.4 (3.7)	*n=412* 30.2 (3.6)	*n=582* 29.3 (4.3)	<0.001
Pre-Pregnancy BMI (kg/m2) **	*n=256* 22.6 (2.9)	*n=391* 22.5 (3.4)	*n=534* 22.7 (3.4)	0.6
**Categorical Variables - n (%)**				
Ethnicity	*n=267*	*n=411*	*n=577*	0.4
White	265 (99.3)	406 (98.8)	566 (98.1)	
Social Class	*n=261*	*n=409*	*n=568*	
I (highest; professional)	59 (22.6)	89 (21.8)	109 (19.2)	
ii	127 (48.7)	198 (48.4)	274 (48.2)	
iii (non-manual)	54 (20.7)	74 (18.1)	141 (24.8)	0.2
iii (manual)	17 (6.5)	37 (9.1)	34 (6.0)	
iv & v (lowest; unskilled manual)	4 (1.5)	11 (2.7)	10 (1.8)	
Education	*n=269*	*n=411*	*n=580*	0.4
University Level or above	66 (24.5)	82 (20.0)	124 (21.4)	
Single Parent household	*n=269*	*n=412*	*n=578*	<0.001
Yes	1 (0.4)	3 (0.7)	24 (4.2)	
Smoked during pregnancy **	*n=268*	*n=412*	*n=581*	1.0
Yes	101 (38.0)	152 (37.0)	2013 (36.7)	

* SD - Standard Deviation

** Relates to the first child in some women and second in others

For peer review only - http://bmjopen.bmj.com/site/about/guidelines.xhtml

TABLE 3 – Distribution of pre-pregnancy BMI and outcomes at research clinic assessment (2009-11) in the full cohortwith IPI

		Inter-pregnancy interval n=1268				
		≤15 months		16-27 months		>27 months
	n=	n=271	n=	n=415	n=	n=582
**Mean (Standard deviation)**						
Pre-pregnancy BMI (kg/m2)	260	22.6(2.8)	399	22.5(3.4)	553	22.7(3.4)
BMI (kg/m2)	203	25.9(4.5)	313	25.8(4.7)	433	26.1(4.8)
Waist (cm)	203	82.6(11.4)	313	82.6(11.2)	433	83.3(11.2)
SBP (mm hg)	202	118.1(12.5)	304	118(12.3)	424	117.4(12.5)
DBP (mm hg)	202	71.7(8.3)	304	71.7(8.5)	424	71.4(8.1)
Heart rate	202	84.3(11.1)	304	83.7(10.7)	424	83.6(11)
Fat Mass (kg)	204	26(10.1)	314	25.9(9.9)	428	26.3(10.1)
Avg. Arterial Distensibility (mm)	204	0.5(0.1)	315	0.5(0.1)	430	0.5(0.1)
Common Carotid Intima (mm)	204	0.6(0.1)	315	0.6(0.1)	430	0.6(0.1)
Cholesterol (mmol/l)	194	4.9(0.8)	303	5(0.9)	410	4.9(0.9)
High-density Lipoprotein (mmol/l)	194	1.5(0.4)	303	1.5(0.4)	410	1.5(0.4)
Non-HDL cholesterol (mmol/l)	194	3.4(0.9)	303	3.5(0.9)	410	3.4(0.9)
**Median (Inter-quartile range)**						
Insulin (mu/1)	193	4.3(3.2,6.4)	301	4.3(3,6.3)	408	4.6(3.3,6.8)
Pro-Insulin (pu/l)	194	5(3.6,7.3)	302	4.9(3.5,6.6)	409	5.1(3.6,7.9)
Triglyceride (mmol/l)	194	0.8(0.6,1.1)	303	0.9(0.7,1.2)	410	0.9(0.7,1.1)
C-reactive protein (mg/l)	194	0.9(0.4,1.9)	303	0.9(0.5,2)	410	1(0.5,2.5)
Glucose (mmol/l)	194	5.2(4.9,5.4)	303	5.2(4.9,5.4)	410	5.1(4.9,5.4)

For peer review only - http://bmjopen.bmj.com/site/about/guidelines.xhtml

Association between inter-pregnancy intervalIPI and CVD risk factors

Crude associations of inter-pregnancy intervalIPI with outcomes are presented in table 4 and 5. We found no evidence that women with either short or long inter-pregnancy intervalIPI had more adverse cardiovascular risk factors compared with women with an inter-pregnancy intervalIPI between 16-27 months. There was some suggestion that women with both long and short inter-pregnancy intervalIPI had a more favourable lipid profile compared with women whose inter-pregnancy intervalIPI was 16-27 months, however the differences were small in magnitude and imprecisely estimated, with the 95% confidence interval including the null value.

Adjustment for potential confounders or for potential mediation by BMI at outcome assessment did not change results (tables 4, 5 and web table 43). These findings are similar to the results when the analysis was restricted to complete cases (web table 54 and 65). The results were also similar when the women were stratified according to whether the ALSPAC child was the first or second pregnancy (results not shown – available from authors on request).

For peer review only

TABLE 4 – Unadjusted and confounder adjusted analysis of the association between inter-pregnancy interval and blood based cardiovascular risk factors using multivariate multiply imputed data (N=1268)

		Inter-pregnancy interval				
		≤15 months		16-27 months	>27 months	
~~IBI (months)~~	Analysis Model Number	β* (95% CI)	P-value	Ref Group	β* (95% CI)	P-value
Cholesterol (mmol/l)	Model 1[a]	-0.1 (-0.2,0.1)	0.3	0	-0.1 (-0.2,0.03)	0.1
	Model 2[b]	-0.1 (-0.3,0.1)	0.2	0	-0.05 (-0.2,0.1)	0.4
HDL (mmol/l)	Model 1[a]	0.03 (-0.04,0.1)	0.4	0	-0.01 (-0.1,0.05)	0.8
	Model 2[b]	0.03 (-0.04,0.1)	0.4	0	0.01 (0,0.1)	0.6
		% Change (95% CI)	P-value	Ref Group	% Change (95% CI)	P-value
Insulin (mu/l)**	Model 1[a]	1 (0.9,1.1)	0.8	0	1.1 (1,1.2)	0.1
	Model 2[b]	1 (0.9,1.1)	0.9	0	1 (1,1.1)	0.4
Pro-Insulin (pmol/l)**	Model 1[a]	1 (0.9,1.1)	0.7	0	1.1 (1,1.1)	0.1
	Model 2[b]	1 (0.9,1.1)	0.8	0	1 (1,1.1)	0.2
Triglyceride (mmol/l)**	Model 1[a]	0.9 (0.9,1)	0.1	0	0.9 (0.9,1)	0.03
	Model 2[b]	0.9 (0.9,1)	0.1	0	0.9 (0.9,1)	0.02
CRP (mg/l)**	Model 1[a]	0.9 (0.8,1.1)	0.4	0	1.1 (0.9,1.2)	0.4
	Model 2[b]	0.9 (0.8,1.1)	0.5	0	1 (0.9,1.2)	0.6
Glucose (mmol/l)**	Model 1[a]	1 (1,1)	0.7	0	1 (1,1)	0.4
	Model 2[b]	1 (1,1)	0.6	0	1 (1,1)	0.3

* Mean difference compared with 16-27 months

** Log transformed

[a] Unadjusted model

[b] Adjusted for maternal age, maternal ethnicity, social class, education, pre-pregnancy BMI, ever smoking during pregnancy

For peer review only

For peer review only - http://bmjopen.bmj.com/site/about/guidelines.xhtml

TABLE 5 - Unadjusted and confounder adjusted analysis of the association between inter-pregnancy interval and non-blood based cardiovascular risk factors using multivariate multiply imputed data (N=1268)

		Inter-pregnancy interval				
	Analysis Model Number	≤15 months		16-27 months Ref	>27 months	
		β* (95% CI)	P-value	Group	β* (95% CI)	P-value
Avg. Arterial Distensibility (mm)	Model 1[a]	-0.0003 (-0.02,0.02)	1	0	0.02 (0.001,0.04)	0.04
	Model 2[b]	0.002 (-0.02,0.02)	0.9	0	0.01 (-0.003,0.03)	0.1
Common Carotid Intima (mm)	Model 1[a]	0.005 (-0.01,0.02)	0.4	0	-0.0008 (-0.01,0.01)	0.9
	Model 2[b]	0.003 (-0.01,0.01)	0.6	0	0.002 (-0.01,0.01)	0.7
Total body fat mass (kg)[c]	Model 1[a]	-0.2 (-1.7,1.3)	0.8	0	0.4 (-0.8,1.7)	0.5
	Model 2[b]	-0.3 (-1.6,0.9)	0.6	0	-0.05 (-1.1,1)	0.9
BMI (kg/m2)	Model 1[a]	0.03 (-0.7,0.8)	0.9	0	0.3 (-0.3,0.9)	0.3
	Model 2[b]	-0.02 (-0.6,0.5)	0.9	0	0 (-0.4,0.5)	0.8
Waist (cm)	Model 1[a]	-0.1 (-1.8,1.6)	0.9	0	0.6 (-0.8,2.1)	0.4
	Model 2[b]	-0.3 (-1.7,1.1)	0.7	0	0.1 (-1.1,1.4)	0.8
Systolic BP (mm hg)	Model 1[a]	0.1 (-1.9,2.2)	0.9	0	-0.6 (-2.3,1.2)	0.5
	Model 2[b]	0.1 (-1.9,2.1)	0.9	0	-0.5 (-2.3,1.2)	0.5
Diastolic BP (mm hg)	Model 1[a]	0.1 (-1.3,1.4)	0.9	0	-0.4 (-1.6,0.8)	0.5
	Model 2[b]	0.1 (-1.2,1.5)	0.9	0	-0.5 (-1.7,0.7)	0.4
Heart Rate	Model 1[a]	0.4 (-1.6,2.4)	0.7	0	0 (-1.7,1.6)	1
	Model 2[b]	0.3 (-1.7,2.4)	0.7	0	0 (-1.6,1.7)	1

* Mean difference compared with 16-27 months

[a] Unadjusted model

[b] Adjusted for age, ethnicity, social class, education, pre-pregnancy BMI, ever smoking during pregnancy

[c] Height and height^2 were included into the model

For peer review only

DISCUSSION

We found no association between short or long ~~inter-pregnancy interval~~IPI and levels of cardiovascular risk factors in a cohort of women with a mean age of 48 years. This is in contrast to our initial hypotheses that shorter ~~inter-pregnancy interval~~IPIs may give a woman's cardiovascular system insufficient time to recover, that longer ~~inter-pregnancy interval~~IPIs may reflect subfertility and that both may be associated with adverse levels of cardiovascular risk factors. We actually found weak evidence that women with both short (≤15 months) and long ~~inter-pregnancy interval~~IPIs (>27) had a more favourable lipid profile (i.e. the opposite of our initial hypotheses) compared with women with an ~~inter-pregnancy interval~~IPI between 16 and 27 months, but the associations were not large, were imprecisely estimated, and were not consistent across all outcomes and may be due to chance.

To our knowledge, this is the first study to examine the relationship between pregnancy interval and subsequent cardiovascular outcomes. Several previous studies have examined the association of parity with cardiovascular outcomes, with most,[6-12] though not all,[14-16] finding that greater parity is related to more adverse risk factors and greater disease risk. A recent study investigated this association in a population of 1.3 million, using the Swedish registry data,[12] and found a J-shaped relationship between parity and CVD risk, with both nulliparous and grand multiparous (≥ 5 births) having elevated risk compared to those women with 2 births. One possible mechanism for this association is that multiparous women are more likely to have births closer together, and the repeating of the cardiovascular challenge within a relatively short amount of time may lead to the effects on a women's metabolic system to be exacerbated and/or be longer lasting. Our findings do not support this hypothesis. Our results therefore suggest that the association of parity with greater CVD risk may be through another mechanism. Possible theories for the mechanisms underlying the greater risk of CVD after 2 children are: 1) other adverse lifestyle factors being adopted as a family size increases; 2) socio-demographic and other characteristics associated with increased risk of CVD also being associated with having more children; 3) adverse metabolic disturbances accumulating over pregnancies.[12]

The strengths of this study include the prospective design; detailed data on reproductive history; the availability of a wide range of objectively-measured CVD risk factors; and the large sample size. We were able to adjust for a range of potential confounding factors, and we restricted our analyses to women who had two live births in order to remove confounding by parity. To the best of our knowledge, we are the first study to examine the association of ~~inter-pregnancy interval~~IPI with cardiovascular risk factors. The findings of this study however should be

For peer review only

considered in light of several limitations. As with other prospective cohort studies there was loss to follow-up, with those attending and completing all questionnaires tending to come from a higher socioeconomic background. This means that the women included in our analysis are not a representative sample of the full ALSPAC cohort; some studies suggest that lack of generalisability does not necessarily result in selection bias [17-20], but we cannot be certain of this from the current analysis and replication of our results in other studies with different distributions of socio-demographic variables would be beneficial. Missing data would bias our results if the association between ~~inter-pregnancy interval~~IPI and cardiovascular risk factors differed in those included in our analyses and those excluded due to missing data. We are unable to test this assumption, but have no reason to suspect that it may be violated. One important consideration when interpreting our results is that we have restricted our analyses to women who had two live births. Whilst we feel that this was a sensible analysis strategy in order to remove confounding by parity, it means that our findings may not generalise to women who had three or more children. We did not find any evidence of any strong differences between women who had either only one birth or three or more births, with women with two live/still births (web table ~~1~~3). ~~Inter-pregnancy interval~~IPI was calculated by subtracting the average gestation period (9 months) from the birth interval, ideally we would have liked to have calculated exact gestation periods for each live birth. We do not, however, believe that this would have biased our findings as we used ~~inter-pregnancy interval~~IPI as a categorical variable and therefore any fluctuations around the average 9 month gestation would not have altered the findings substantially. It is possible that by calculating our IPI in this way we have attenuated our results towards the null. A further limitation of this study is these findings are only generalizable to a largely white European population with a higher socioeconomic status. As the population being studied are still young (mean age at clinic =48), it is possible that the association may emerge at older ages, when inter-individual variability in cardiovascular risk factors becomes greater. Ideally we would have measured pre-pregnancy BMI and smoking during pregnancy for the first pregnancy of all women. Due to our study design, this was not possible. These measurements are from the first pregnancy in some women and the second pregnancy in others. There is therefore the possibility that these measurements are not a reasonable representation of levels in the first pregnancy for all women; this may lead to residual confounding. However given that the associations we observe are null, we do not think this has biased our results.

In conclusion, our results do not support an association between ~~inter-pregnancy interval~~IPI and cardiovascular risk factors, though our findings must be interpreted in light of the large losses to follow-up and limitations in our measurement of IPI.~~.~~ Further studies in other populations with more detailed data on gestational age

For peer review only

at delivery of all pregnancies, in different settings such as low income countries where the social patterning of inter-pregnancy intervalIPI may differ and in older women with greater inter-individual variability in cardiovascular risk factors would provide a more comprehensive understanding of these associations.

For peer review only

For peer review only - http://bmjopen.bmj.com/site/about/guidelines.xhtml

ACKNOWLEDGEMENTS

We are extremely grateful to all the families who took part in this study, the midwives for their help in recruiting them, and the whole ALSPAC team, which includes interviewers, computer and laboratory technicians, clerical workers, research scientists, volunteers, managers, receptionists and nurses. This publication is the work of the authors and DWK will serve as guarantor for the contents of this paper.

COMPETING INTERESTS

None declared

FUNDING

The work was supported by the British Heart Foundation [SP/07/008/24066], the UK Medical Research Council [0701594 to A.F and G1002375 to LH] and the Wellcome Trust (and WT099874MA to D.W.K). AF, DAL and LDH work in a unit that receives funding from the UK Medical Research Council and the University of Bristol. The UK Medical Research Council ([074882], the Wellcome Trust [WT076467] and the University of Bristol provide core funding support for ALSPAC.

AUTHOR CONTRIBUTIONS

DAL, LH and AF conceived the study idea; DWK, LH and AF developed the analysis plan; DWK undertook the analysis under the supervision of LH and AF; DWK wrote the first draft of the paper and collated co-author feedback; and all authors contributed to the critical review and final version.

For peer review only - http://bmjopen.bmj.com/site/about/guidelines.xhtml

REFERENCES

1 N. Sattar, Do pregnancy complications and CVD share common antecedents? *Atheroscler Suppl* 2004;**5:**3-7

2 A. Wendt, C. M. Gibbs, S. Peters, et al., Impact of increasing inter-pregnancy interval on maternal and infant health. *Paediatric and perinatal epidemiology* 2012;**26 Suppl 1:**239-58

3 C. G. Solomon, F. B. Hu, A. Dunaif, et al., Menstrual cycle irregularity and risk for future cardiovascular disease. *The Journal of clinical endocrinology and metabolism* 2002;**87:**2013-7

4 N. I. Parikh, S. Cnattingius, M. A. Mittleman, et al., Subfertility and risk of later life maternal cardiovascular disease. *Human reproduction* 2012;**27:**568-75

5 A. Fraser, C. Macdonald-Wallis, K. Tilling, et al., Cohort Profile: The Avon Longitudinal Study of Parents and Children: ALSPAC mothers cohort. *Int J Epidemiol* 2012;97-110

6 F. Atsma, M. L. Bartelink, D. E. Grobbee, et al., Reproductive factors, metabolic factors, and coronary artery calcification in older women. *Menopause* 2008;**15:**899-904

7 J. M. Catov, A. B. Newman, K. Sutton-Tyrrell, et al., Parity and cardiovascular disease risk among older women: how do pregnancy complications mediate the association? *Ann Epidemiol* 2008;**18:**873-9

8 L. G. Gallagher, L. B. Davis, R. M. Ray, et al., Reproductive history and mortality from cardiovascular disease among women textile workers in Shanghai, China. *International Journal of Epidemiology* 2011;**40:**1510-1518

9 A. Green, V. Beral and K. Moser, Mortality in Women in Relation to Their Childbearing History. *Brit Med J* 1988;**297:**391-395

10 D. H. Jaffe, Z. Eisenbach and O. Manor, The effect of parity on cause-specific mortality among married men and women. *Matern Child Health J* 2011;**15:**376-85

11 R. B. Ness, T. Harris, J. Cobb, et al., Number of pregnancies and the subsequent risk of cardiovascular disease. *N Engl J Med* 1993;**328:**1528-33

12 N. I. Parikh, S. Cnattingius, P. W. Dickman, et al., Parity and risk of later-life maternal cardiovascular disease. *American heart journal* 2010;**159:**215-221 e6

13 P. Royston, Multiple imputation of missing values. *Stata J* 2004;**4:**227-241

14 H. S. Chang, N. Odongua, H. Ohrr, et al., Reproductive risk factors for cardiovascular disease mortality among postmenopausal women in Korea: the Kangwha Cohort Study, 1985-2005. *Menopause* 2011;**18:**1205-12

15 G. A. Colditz, W. C. Willett, M. J. Stampfer, et al., A prospective study of age at menarche, parity, age at first birth, and coronary heart disease in women. *Am J Epidemiol* 1987;**126:**861-70

16 K. Steenland, C. Lally and M. Thun, Parity and coronary heart disease among women in the American Cancer Society CPS II population. *Epidemiology* 1996;**7:**641-3

17 A. J. Sogaard, R. Selmer, E. Bjertness, et al., The Oslo Health Study: The impact of self-selection in a large, population-based survey. *International journal for equity in health* 2004;**3:**3

18 A. J. Van Loon, M. Tijhuis, H. S. Picavet, et al., Survey non-response in the Netherlands: effects on prevalence estimates and associations. *Ann Epidemiol* 2003;**13:**105-10

19 R. M. Nilsen, S. E. Vollset, H. K. Gjessing, et al., Self-selection and bias in a large prospective pregnancy cohort in Norway. *Paediatric and perinatal epidemiology* 2009;**23:**597-608

20 E. A. Nohr, M. Frydenberg, T. B. Henriksen, et al., Does low participation in cohort studies induce bias? *Epidemiology* 2006;**17:**413-8

For peer review only - http://bmjopen.bmj.com/site/about/guidelines.xhtml

FIGURES LEGENDS

Figure 1 – Flow-chart of selection of women for analysis

For peer review only

For peer review only - http://bmjopen.bmj.com/site/about/guidelines.xhtml

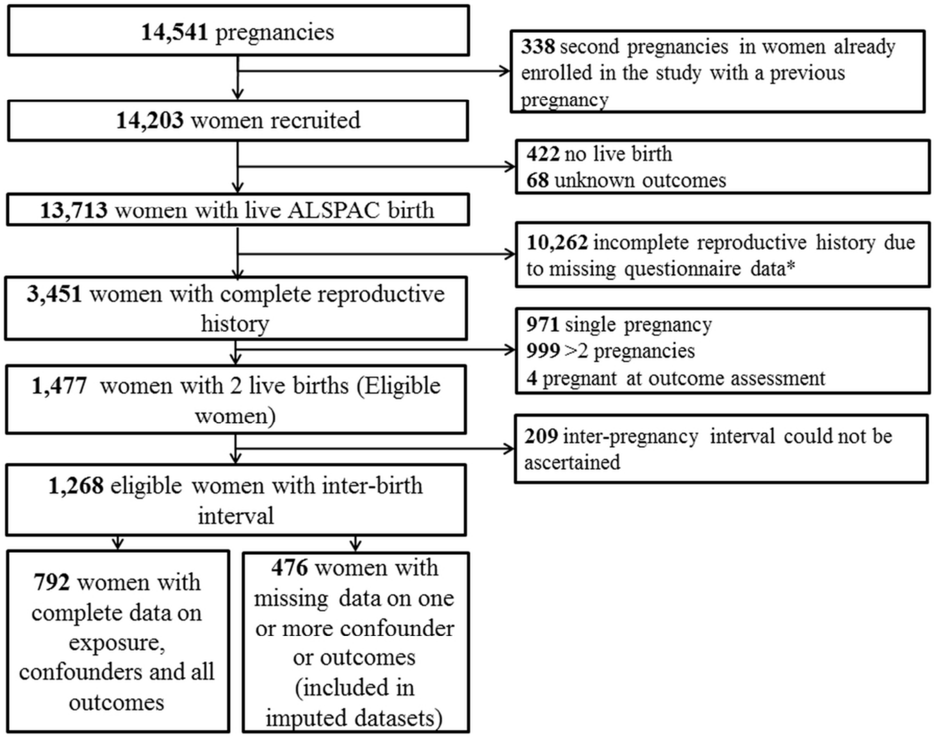

119x90mm (300 x 300 DPI)

Web table 1 – Distributions of imputed characteristics (% or mean (standard error)) in the imputation datasets and in the complete case sample (i.e. non-imputed)

Imputed Variables		Missing (n)	% data imputed	Imputed Data	Non-imputed
**Exposure**					
Inter pregnancy interval		0	0%	N/A	44.85 (0.84)
**Confounders**					
Pre-pregnancy BMI		61	5%	22.6 (0.1)	22.6 (0.1)
Height		17	1%	164.7 (0.2)	164.7 (0.2)
Single parent household		4	0.3%	2%	2%
Education	< O-Level	3	0.2%	13%	13%
	O-Level			34%	34%
	A-Level			32%	32%
	> A-Level			22%	22%
Social Class	i	25	2%	21%	21%
	ii			48%	48%
	iii (non-manual)			22%	22%
	iii (manual)			7%	7%
	iv & v			2%	2%
Ethnicity - White		8	0.6%	99%	99%
Ever Smoked		2	0.2%	37%	37%
Age		0	0%	27.9 (0.1)	27.9 (0.1)
**Outcomes at follow-up clinic**					
Avg. Arterial Distensibility (mm)		323	26%	0.5 (0.004)	0.5 (0.004)
Common Carotid Intima (mm)		323	26%	0.6 (0.002)	0.6 (0.002)
Waist (cm)		324	26%	83.1 (0.3)	82.9 (0.4)
SBP (mm hg)		343	27%	117.9 (0.4)	117.7 (0.4)
Fat Mass (kg)		327	26%	26.3 (293.9)	26.1 (327.9)
BMI (kg/m2)		324	26%	26.1 (0.1)	25.9 (0.2)
DBP (mm hg)		343	27%	71.7 (0.3)	71.5 (0.3)
Heart rate		343	27%	83.7 (0.4)	83.8 (0.4)
Cholesterol (mmol/l)		365	29%	4.9 (0.03)	4.9 (0.03)
HDL (mmol/l)		365	29%	1.5 (0.01)	1.5 (0.01)
LDL (mmol/l)		365	29%	3 (0.03)	3 (0.03)
Non-HDL cholesterol (mmol/l)		365	29%	3.4 (0.03)	3.4 (0.03)
Insulin (mu/1) (log)		370	29%	1.5 (0.02)	1.5 (0.02)
Pro-Insulin (pu/l) (log)		367	29%	1.7 (0.02)	1.7 (0.02)
Triglyceride (mmol/l) (log)		365	29%	-0.1 (0.01)	-0.1 (0.01)
CRP(mg/l) (log)		365	29%	0.1 (0.04)	0 (0.04)
Glucose (mmol/l) (log)		365	29%	1.6 (0.004)	1.6 (0.004)

For peer review only - http://bmjopen.bmj.com/site/about/guidelines.xhtml

Web table 2 – Comparison of inter-pregnancy interval between women included in the complete case analysis (n=792) versus women with missing data (n=476)

	All included women n=1268		
	Missing data n=476	Complete Case n=792	P-value
Inter-pregnancy interval (months)			
≤15	100 (21.0)	171 (21.6)	
16-27	146 (31.7)	269 (34.0)	0.37
>27	230 (48.3)	352 (44.4)	

For peer review only

Web table 3 – Characteristics of eligible women (with two live births) compared to women with one or three or more live/still births, with full reproductive history n=3447

		No. of births						
		1 birth (n=972)		2 births (n=1479)		3 or more births (n=1000)		P-value
Confounder Variables								
Ethnicity - White n (%)		n=961	948 (98.7)	n=1469	1449 (98.6)	n=987	973 (98.6)	1.0
Social Class n (%)	i		177 (18.9)		297 (20.5)		190 (20.0)	
	ii		471 (50.2)		693 (47.9)		437 (46.1)	
	iii (non-manual)	n=938	224 (23.9)	n=1448	326 (22.5)	n=949	188 (19.8)	<0.001
	iii (manual)		54 (5.8)		100 (6.9)		104 (12.0)	
	iv & v		12 (1.3)		32 (2.2)		30 (3.2)	
Education (University Level or above) n (%)		n=962	212 (22.0)	n=1475	313 (21.2)	n=988	217 (22.0)	0.9
Single Parent Household n (%)		n=946	30 (3.2)	n=1474	35 (2.4)	n=983	37 (3.8)	0.1
Ever smoked n (%)		n=969	380 (39.2)	n=1476	557 (37.7)	n=996	432 (43.4)	0.02
Age mean (SD)*		n=972	29.3 (4.3)	n=1479	29.9 (4.1)	n=1000	30.0 (4.8)	0.0004
Pre-Preg BMI (kg/m2)* - median (IQR)		n=923	21.9 (20.4,24.1)	n=1403	22.1 (20.4,24.1)	n=931	22.1 (20.5,24.1)	0.81
Outcome Variables - median (IQR)								
BMI (kg/m2)*		n=730	25.2 (22.6,28.5)	n=1095	25 (22.6,29.3)	n=705	25.9 (22.9,29.3)	0.003
Waist (cm)*		n=730	81.3 (74.7,89.3)	n=1095	81 (74.8,92.3)	n=705	83.1 (75.7,92.3)	0.0003
SBP (mm hg)*		n=714	116.8 (109.8,126)	n=1071	115.3 (109,125.5)	n=691	117 (110.3,125.5)	0.2
DBP (mm hg)*		n=714	71 (66,76.8)	n=1071	70.3 (65.5,76.3)	n=691	71.3 (66.3,76.3)	1.0
Pulse*		n=714	82.5 (76,89.5)	n=1071	83 (76.8,91.5)	n=691	83.8 (77,91.5)	0.01
Fat Mass (kg)*		n=724	25.3 (19.1,32)	n=1091	24.2 (18.9,34.2)	n=704	25.9 (18.9,34.2)	0.01
Avg. Arterial Distensibility (mm)*		n=716	0.5 (0.4,0.6)	n=1069	0.5 (0.4,0.6)	n=691	0.5 (0.4,0.6)	0.5
Common Carotid Intima (mm)*		n=719	0.6 (0.5,0.6)	n=1069	0.6 (0.5,0.6)	n=691	0.6 (0.5,0.6)	1.0
Cholesterol (mmol/l)*		n=692	4.8 (4.3,5.4)	n=1043	4.9 (4.3,5.5)	n=676	4.9 (4.4,5.5)	0.2
High-density Lipoprotein (mmol/l)*		n=692	1.5 (1.2,1.7)	n=1043	1.5 (1.2,1.7)	n=676	1.4 (1.2,1.7)	0.1
Non-HDL cholesterol (mmol/l)*		n=692	3.3 (2.7,4)	n=1043	3.4 (2.8,4)	n=676	3.5 (2.9,4)	0.1
Very-low density lipoprotein (mmol/l)*		n=692	0.4 (0.3,0.5)	n=1043	0.4 (0.3,0.6)	n=676	0.4 (0.3,0.6)	0.03
Insulin (mu/l)**		n=692	4.7 (3.3,6.3)	n=1038	4.5 (3.2,6.8)	n=674	4.7 (3.2,6.8)	0.2
Pro-insulin (pu/l)**		n=692	5 (3.7,7.2)	n=1041	5 (3.6,8)	n=675	5.3 (3.7,8)	0.2
Triglyceride (mmol/l)**		n=692	0.9 (0.7,1.2)	n=1043	0.9 (0.7,1.2)	n=676	0.9 (0.7,1.2)	0.1
CRP (mg/l)**		n=692	0.9 (0.5,2)	n=1043	0.9 (0.4,2.2)	n=676	1 (0.5,2.2)	0.5
Glucose (mmol/l)**		n=692	5.2 (4.9,5.5)	n=1043	5.1 (4.9,5.5)	n=676	5.2 (4.9,5.5)	0.3

* linear regression for p-value

** log transformed for linear regression

For peer review only - http://bmjopen.bmj.com/site/about/guidelines.xhtml

Web table 4 - Confounder and mediator adjusted analysis of the association between inter-pregnancy interval and cardiovascular risk factors using multivariate multiply imputed data (N=1268)

	Inter-pregnancy interval [a]				
	≤15 months		16-27 months	>27 months	
	β* (95% CI)	P-value	Ref Group	β* (95% CI)	P-value
Avg. Arterial Distensibility (mm)	-0.001 (-0.02,0.02)	0.9	0	0.01 (-0.004,0.03)	0.1
Common Carotid Intima (mm)	0.004 (-0.01,0.02)	0.5	0	0.002 (-0.01,0.01)	0.7
Waist (cm)	-0.1 (-1,0.7)	0.8	0	0.1 (-0.7,0.9)	0.8
Systolic BP (mm hg)	0.3 (-1.8,2.3)	0.8	0	-0.4 (-2.2,1.3)	0.6
Diastolic BP (mm hg)	0.2 (-1.1,1.6)	0.8	0	-0.5 (-1.6,0.6)	0.4
Heart Rate	0.4 (-1.5,2.3)	0.7	0	-0.1 (-1.8,1.7)	1.0
Cholesterol (mmol/l)	-0.1 (-0.3,0.04)	0.1	0	-0.1 (-0.2,0.1)	0.4
HDL (mmol/l)	0.02 (-0.05,0.1)	0.6	0	0.02 (-0.04,0.1)	0.6
	% Change (95% CI)	P-value	Ref Group	% Change (95% CI)	P-value
Insulin (mu/l)**	1 (0.9,1.1)	1.0	0	1 (0.9,1.1)	0.6
Pro-Insulin (pmol/l)**	1 (1,1.1)	0.4	0	1.1 (1,1.1)	0.2
Triglyceride (mmol/l)**	0.9 (0.9,1)	0.1	0	0.9 (0.9,1)	0.02
CRP (mg/l)**	0.9 (0.8,1.1)	0.6	0	1 (0.9,1.2)	0.7
Glucose (mmol/l)**	1 (1,1)	0.6	0	1 (1,1)	0.3

* Mean difference compared with 16-27 months

** Log transformed

[a] Adjusted for age, ethnicity, social class, education, pre-pregnancy BMI, ever smoking during pregnancy and BMI at follow-up clinic

For peer review only

Web table 5 – Unadjusted, confounder and mediator adjusted analysis of the association between inter-pregnancy interval and blood based cardiovascular risk factors using complete case data (N=792)

	Analysis Model Number	≤15 months β* (95% CI)	P-value	16-27 months Ref Group	>27 months β* (95% CI)	P-value
Cholesterol (mmol/l)	Model 1[a]	-0.2(-0.3,-0.004)	0.04	0	-0.2(-0.3,-0.01)	0.03
	Model 2[b]	-0.2(-0.3,-0.02)	0.03	0	-0.1(-0.2,0.1)	0.3
	Model 3[c]	-0.2(-0.4,-0.03)	0.02	0	-0.1(-0.2,0.1)	0.3
HDL (mmol/l)	Model 1[a]	0.02(-0.1,0.1)	0.6	0	0(-0.1,0.1)	0.9
	Model 2[b]	0.02(-0.1,0.1)	0.7	0	0.02(-0.04,0.1)	0.4
	Model 3[c]	0.02(-0.04,0.1)	0.5	0	0.03(-0.03,0.1)	0.4
		% Change (95% CI)	P-value	Ref Group	% Change (95% CI)	P-value
Insulin (mu/l)**	Model 1[a]	1(0.9,1.2)	0.6	0	1.1(1,1.2)	0.2
	Model 2[b]	1(0.9,1.2)	0.6	0	1(0.9,1.2)	0.4
	Model 3[c]	1(0.9,1.1)	0.9	0	1(0.9,1.1)	0.4
Pro-Insulin (pmol/l)**	Model 1[a]	1(0.9,1.2)	0.4	0	1.1(1,1.2)	0.1
	Model 2[b]	1(0.9,1.1)	0.4	0	1.1(1,1.2)	0.1
	Model 3[c]	1(0.9,1.1)	0.6	0	1.1(1,1.1)	0.1
Triglyceride (mmol/l)**	Model 1[a]	0.9(0.9,1)	0.1	0	0.9(0.8,1)	0.003
	Model 2[b]	0.9(0.9,1)	0.1	0	0.9(0.8,1)	0.003
	Model 3[c]	0.9(0.8,1)	0.02	0	0.9(0.8,1)	0.001
CRP (mg/l)**	Model 1[a]	1(0.8,1.2)	0.6	0	1(0.8,1.2)	0.9
	Model 2[b]	1(0.8,1.2)	0.7	0	1(0.8,1.2)	0.8
	Model 3[c]	0.9(0.8,1.1)	0.4	0	1(0.8,1.1)	0.7
Glucose (mmol/l)**	Model 1[a]	1(1,1)	0.6	0	1(1,1)	0.5
	Model 2[b]	1(1,1)	0.5	0	1(1,1)	0.4
	Model 3[c]	1(1,1)	0.4	0	1(1,1)	0.4

* Mean difference compared with 16-27 months

** Log transformed

[a] Unadjusted model

[b] Adjusted for age, ethnicity, social class, education, pre-pregnancy BMI, ever smoking during pregnancy

[c] Adjusted for age, ethnicity, social class, education, pre-pregnancy BMI, ever smoking during pregnancy and BMI at follow-up clinic

For peer review only - http://bmjopen.bmj.com/site/about/guidelines.xhtml

Web table 6 - Unadjusted, confounder and mediator analysis of the association between inter-pregnancy interval and non-blood based cardiovascular risk factors using complete case data (N=792)

	Analysis Model Number	Inter-pregnancy interval ≤15 months β* (95% CI)	≤15 months P-value	16-27 months Ref Group	>27 months β* (95% CI)	>27 months P-value
Avg. Arterial Distensibility (mm)	Model 1[a]	-0.005(-0.03,0.02)	0.7	0	0.02(0.001,0.04)	0.04
	Model 2[b]	-0.003(-0.02,0.02)	0.8	0	0.01(-0.01,0.03)	0.2
	Model 3[c]	-0.002(-0.02,0.02)	0.9	0	0.01(-0.01,0.03)	0.2
Common Carotid Intima (mm)	Model 1[a]	0.01(-0.005,0.02)	0.2	0	-0.002(-0.01,0.01)	0.8
	Model 2[b]	0.01(-0.01,0.02)	0.3	0	0.003(-0.01,0.01)	0.6
	Model 3[c]	0.01(-0.01,0.02)	0.4	0	0.003(-0.01,0.01)	0.6
Total body fat mass (kg)[d]	Model 1[a]	0.2(-1.7,2.1)	0.8	0	0.3(-1.2,1.9)	0.7
	Model 2[b]	0.1(-1.3,1.4)	0.9	0	0(-1.1,1.2)	1.0
BMI (kg/m2)	Model 1[a]	0.3(-0.6,1.2)	0.5	0	0.3(-0.5,1)	0.5
	Model 2[b]	0.3(-0.3,0.8)	0.3	0	0.1(-0.4,0.6)	0.7
Waist (cm)	Model 1[a]	0.6(-1.5,2.7)	0.6	0	0.8(-1,2.5)	0.4
	Model 2[b]	0.5(-1.1,2.1)	0.5	0	0.5(-0.8,1.8)	0.5
	Model 3[c]	-0.1(-1.1,0.9)	0.8	0	0.3(-0.6,1.1)	0.5
Systolic BP (mm hg)	Model 1[a]	0.8(-1.6,3.2)	0.5	0	-0.6(-2.6,1.4)	0.6
	Model 2[b]	0.6(-1.7,3)	0.6	0	-0.7(-2.7,1.3)	0.5
	Model 3[c]	0.3(-1.9,2.6)	0.8	0	-0.8(-2.7,1.1)	0.4
Diastolic BP (mm hg)	Model 1[a]	0.3(-1.3,1.9)	0.7	0	-0.5(-1.9,0.8)	0.4
	Model 2[b]	0.3(-1.3,1.8)	0.7	0	-0.7(-2,0.7)	0.3
	Model 3[c]	0.1(-1.5,1.6)	0.9	0	-0.7(-2,0.6)	0.3
Heart Rate	Model 1[a]	0.2(-1.9,2.3)	0.8	0	-0.3(-2,1.5)	0.7
	Model 2[b]	0.1(-2,2.2)	0.9	0	-0.3(-2.1,1.5)	0.7
	Model 3[c]	-0.1(-2.1,2)	1.0	0	-0.4(-2.1,1.4)	0.7

* Mean difference compared with 16-27 months

[a] Unadjusted model

[b] Adjusted for l age, l ethnicity, social class, education, pre-pregnancy BMI, ever smoking during pregnancy

[c] Adjusted for age, ethnicity, social class, education, pre-pregnancy BMI, ever smoking during pregnancy and BMI at follow-up clinic

[d] Height and height2 were included into the model

For peer review only

STROBE 2007 (v4) Statement—Checklist of items that should be included in reports of *cohort studies*

Section/Topic	Item #	Recommendation	Reported on page #
**Title and abstract**	1	(*a*) Indicate the study's design with a commonly used term in the title or the abstract	1
		(*b*) Provide in the abstract an informative and balanced summary of what was done and what was found	3
**Introduction**			
Background/rationale	2	Explain the scientific background and rationale for the investigation being reported	5
Objectives	3	State specific objectives, including any prespecified hypotheses	5
**Methods**			
Study design	4	Present key elements of study design early in the paper	6
Setting	5	Describe the setting, locations, and relevant dates, including periods of recruitment, exposure, follow-up, and data collection	6-8
Participants	6	(*a*) Give the eligibility criteria, and the sources and methods of selection of participants. Describe methods of follow-up	6-8 & figure 1
		(*b*) For matched studies, give matching criteria and number of exposed and unexposed	n/a
Variables	7	Clearly define all outcomes, exposures, predictors, potential confounders, and effect modifiers. Give diagnostic criteria, if applicable	6-8
Data sources/ measurement	8*	For each variable of interest, give sources of data and details of methods of assessment (measurement). Describe comparability of assessment methods if there is more than one group	*6-8*
Bias	9	Describe any efforts to address potential sources of bias	17-18 & web tables
Study size	10	Explain how the study size was arrived at	6-9 & figure 1
Quantitative variables	11	Explain how quantitative variables were handled in the analyses. If applicable, describe which groupings were chosen and why	8
Statistical methods	12	(*a*) Describe all statistical methods, including those used to control for confounding	8-9
		(*b*) Describe any methods used to examine subgroups and interactions	8-9
		(*c*) Explain how missing data were addressed	8-9
		(*d*) If applicable, explain how loss to follow-up was addressed	17
		(*e*) Describe any sensitivity analyses	8-9
**Results**			

For peer review only

Participants	13*	(a) Report numbers of individuals at each stage of study—eg numbers potentially eligible, examined for eligibility, confirmed eligible, included in the study, completing follow-up, and analysed	9 & Figure 1
		(b) Give reasons for non-participation at each stage	9 & Figure 1
		(c) Consider use of a flow diagram	Figure 1
Descriptive data	14*	(a) Give characteristics of study participants (eg demographic, clinical, social) and information on exposures and potential confounders	10 & 12
		(b) Indicate number of participants with missing data for each variable of interest	Web table
		(c) Summarise follow-up time (eg, average and total amount)	13
Outcome data	15*	Report numbers of outcome events or summary measures over time	13
Main results	16	(*a*) Give unadjusted estimates and, if applicable, confounder-adjusted estimates and their precision (eg, 95% confidence interval). Make clear which confounders were adjusted for and why they were included	
		(*b*) Report category boundaries when continuous variables were categorized	15 & 16
		(*c*) If relevant, consider translating estimates of relative risk into absolute risk for a meaningful time period	
Other analyses	17	Report other analyses done—eg analyses of subgroups and interactions, and sensitivity analyses	14 & web tables
**Discussion**			
Key results	18	Summarise key results with reference to study objectives	17
**Limitations**			
Interpretation	20	Give a cautious overall interpretation of results considering objectives, limitations, multiplicity of analyses, results from similar studies, and other relevant evidence	17-18
Generalisability	21	Discuss the generalisability (external validity) of the study results	17-18
**Other information**			
Funding	22	Give the source of funding and the role of the funders for the present study and, if applicable, for the original study on which the present article is based	19

*Give information separately for cases and controls in case-control studies and, if applicable, for exposed and unexposed groups in cohort and cross-sectional studies.

Note: An Explanation and Elaboration article discusses each checklist item and gives methodological background and published examples of transparent reporting. The STROBE checklist is best used in conjunction with this article (freely available on the Web sites of PLoS Medicine at http://www.plosmedicine.org/, Annals of Internal Medicine at http://www.annals.org/, and Epidemiology at http://www.epidem.com/). Information on the STROBE Initiative is available at www.strobe-statement.org.

For peer review only
